# Supplementary material for: Comprehensive identification and co-expression analysis of long non-coding RNAs across eight timepoints of Schistosoma mansoni life cycle
Source: Mem Inst Oswaldo Cruz. 2026 Mar 9;121:e250114. doi: 10.1590/0074-02760250114 (PMC12971023; doi:10.1590/0074-02760250114)
Supplement: Supplementary material [file 1678-8060-mioc-121-e250114-s1.pdf]

Supplementary data presenting differential expression analyses (DESeq2) of the identified long non-coding RNAs (lncRNAs) in Figs 1-2, and the co-expression sub-networks from additional weighted gene co-expression network analysis (WGCNA) modules containing hub lncRNAs in Figs 3-5. Detailed information on the RNA-seq libraries used and novel identified lncRNAs is provided in Tables I-II, respectively.

TABLE I

Code of the RNA-seq libraries used for the analyses and the corresponding samples from which they were extracted

BioProject PRJEB32839	
Library	*Schistosoma mansoni* samples
ERR11178266	X_Eggs_R1
ERR11178276	X_Eggs_R2
ERR11178286	X_Eggs_R3
ERR11178295	X_Eggs_R4
ERR11178304	X_Eggs_R5
ERR11178313	X_Miracidia_R1
ERR11178322	X_Miracidia_R2
ERR11178330	X_Miracidia_R3
ERR11178268	X_Miracidia_R4
ERR11178278	X_Miracidia_R5
ERR11178288	X_1d_Sporocysts_R1
ERR11178297	X_1d_Sporocysts_R2
ERR11178306	X_1d_Sporocysts_R3
ERR11178315	X_1d_Sporocysts_R4
ERR11178264	X_1d_Sporocysts_R5
ERR11178332	X_5d_Sporocysts_R1
ERR11178270	X_5d_Sporocysts_R2
ERR11178280	X_5d_Sporocysts_R3
ERR11178290	X_5d_Sporocysts_R4
ERR11178299	X_5d_Sporocysts_R5
ERR11178327	X_32d_Sporocysts_R1
ERR11178336	X_32d_Sporocysts_R2
ERR11178341	X_32d_Sporocysts_R3
ERR11178345	X_32d_Sporocysts_R4
ERR11178342	X_32d_Sporocysts_R5
ERR11178320	X_Cercariae_R1
ERR11178328	X_Cercariae_R2
ERR11178337	X_Cercariae_R3
ERR11178265	X_Cercariae_R4
ERR11178275	X_Cercariae_R5
ERR11178305	X_2d_Somules_R1
ERR11178314	X_2d_Somules_R2
ERR11178323	X_2d_Somules_R3
ERR11178281	X_2d_Somules_R4
ERR11178291	X_2d_Somules_R5
ERR11178331	F_26d_Juveniles_R1
ERR11178269	F_26d_Juveniles_R2
ERR11178279	F_26d_Juveniles_R3
ERR11178307	M_26d_Juveniles_R1
ERR11178316	M_26d_Juveniles_R2
ERR11178324	M_26d_Juveniles_R3

TABLE II

Parameters of the 1,082 novel lncRNAs identified in *Schistosoma mansoni*, including the chromosome where they are located, software used to generate the file, start and end positions, strand orientation (forward [+] or reverse [-]), gene IDs, and transcript IDs

Chromosme	Source	Start	End	Strand	Gene	Transcript
SM_V10_1	StringTie	2095242	2096113	-	MSTRG.52	transcript_id “MSTRG.52.3”
SM_V10_1	StringTie	2095245	2096282	-	MSTRG.52	transcript_id “MSTRG.52.4”
SM_V10_1	StringTie	2095302	2096390	-	MSTRG.52	transcript_id “MSTRG.52.5”
SM_V10_1	StringTie	2124900	2132856	+	MSTRG.53	transcript_id “MSTRG.53.1”
SM_V10_1	StringTie	2686034	2686595	-	MSTRG.63	transcript_id “MSTRG.63.1”
SM_V10_1	StringTie	2720927	2721488	+	MSTRG.64	transcript_id “MSTRG.64.1”
SM_V10_1	StringTie	2989250	2992511	-	MSTRG.69	transcript_id “MSTRG.69.1”
SM_V10_1	StringTie	4021858	4024424	+	MSTRG.89	transcript_id “MSTRG.89.1”
SM_V10_1	StringTie	5750655	5751292	-	MSTRG.108	transcript_id “MSTRG.108.1”
SM_V10_1	StringTie	6606312	6606600	-	MSTRG.121	transcript_id “MSTRG.121.1”
SM_V10_1	StringTie	6622071	6622462	-	MSTRG.122	transcript_id “MSTRG.122.1”
SM_V10_1	StringTie	7354593	7354867	+	MSTRG.136	transcript_id “MSTRG.136.1”
SM_V10_1	StringTie	8541343	8542068	+	MSTRG.143	transcript_id “MSTRG.143.1”
SM_V10_1	StringTie	9719435	9719773	+	MSTRG.156	transcript_id “MSTRG.156.1”
SM_V10_1	StringTie	9727974	9729231	+	MSTRG.157	transcript_id “MSTRG.157.1”
SM_V10_1	StringTie	10045381	10045894	-	MSTRG.166	transcript_id “MSTRG.166.1”
SM_V10_1	StringTie	10194591	10198483	-	MSTRG.169	transcript_id “MSTRG.169.1”
SM_V10_1	StringTie	10467685	10468017	+	MSTRG.175	transcript_id “MSTRG.175.1”
SM_V10_1	StringTie	10595969	10609673	-	MSTRG.179	transcript_id “MSTRG.179.1”
SM_V10_1	StringTie	11294151	11294856	-	MSTRG.190	transcript_id “MSTRG.190.1”
SM_V10_1	StringTie	11505910	11510441	-	MSTRG.194	transcript_id “MSTRG.194.2”
SM_V10_1	StringTie	11820030	11825356	-	MSTRG.201	transcript_id “MSTRG.201.1”
SM_V10_1	StringTie	11870814	11871218	+	MSTRG.202	transcript_id “MSTRG.202.1”
SM_V10_1	StringTie	12370469	12370747	+	MSTRG.211	transcript_id “MSTRG.211.1”
SM_V10_1	StringTie	12709436	12711368	-	MSTRG.218	transcript_id “MSTRG.218.1”
SM_V10_1	StringTie	13634052	13648405	-	MSTRG.227	transcript_id “MSTRG.227.1”
SM_V10_1	StringTie	14355942	14357903	+	MSTRG.234	transcript_id “MSTRG.234.2”
SM_V10_1	StringTie	14356077	14357903	+	MSTRG.234	transcript_id “MSTRG.234.3”
SM_V10_1	StringTie	14356203	14357903	+	MSTRG.234	transcript_id “MSTRG.234.4”
SM_V10_1	StringTie	14356566	14357903	+	MSTRG.234	transcript_id “MSTRG.234.5”
SM_V10_1	StringTie	14356570	14357903	+	MSTRG.234	transcript_id “MSTRG.234.6”
SM_V10_1	StringTie	14356577	14357903	+	MSTRG.234	transcript_id “MSTRG.234.7”
SM_V10_1	StringTie	14356794	14357903	+	MSTRG.234	transcript_id “MSTRG.234.8”
SM_V10_1	StringTie	14356833	14357903	+	MSTRG.234	transcript_id “MSTRG.234.9”
SM_V10_1	StringTie	14356869	14357903	+	MSTRG.234	transcript_id “MSTRG.234.10”
SM_V10_1	StringTie	14398292	14399770	+	MSTRG.237	transcript_id “MSTRG.237.2”
SM_V10_1	StringTie	14399328	14399770	+	MSTRG.237	transcript_id “MSTRG.237.3”
SM_V10_1	StringTie	14469530	14469782	+	MSTRG.242	transcript_id “MSTRG.242.1”
SM_V10_1	StringTie	14525125	14531568	+	MSTRG.244	transcript_id “MSTRG.244.1”
SM_V10_1	StringTie	14528783	14531568	+	MSTRG.244	transcript_id “MSTRG.244.2”
SM_V10_1	StringTie	14969759	14999474	+	MSTRG.252	transcript_id “MSTRG.252.1”
SM_V10_1	StringTie	15017752	15024480	+	MSTRG.253	transcript_id “MSTRG.253.1”
SM_V10_1	StringTie	15232229	15239371	+	MSTRG.263	transcript_id “MSTRG.263.1”
SM_V10_1	StringTie	16082979	16084764	-	MSTRG.275	transcript_id “MSTRG.275.1”

Chromosme	Source	Start	End	Strand	Gene	Transcript
SM_V10_1	StringTie	16195729	16196219	+	MSTRG.277	transcript_id “MSTRG.277.1”
SM_V10_1	StringTie	16195729	16205257	+	MSTRG.277	transcript_id “MSTRG.277.2”
SM_V10_1	StringTie	16578036	16580679	+	MSTRG.281	transcript_id “MSTRG.281.1”
SM_V10_1	StringTie	17649449	17684375	-	MSTRG.297	transcript_id “MSTRG.297.1”
SM_V10_1	StringTie	18606684	18607387	-	MSTRG.314	transcript_id “MSTRG.314.1”
SM_V10_1	StringTie	19425935	19426171	-	MSTRG.317	transcript_id “MSTRG.317.1”
SM_V10_1	StringTie	19719235	19719759	-	MSTRG.320	transcript_id “MSTRG.320.1”
SM_V10_1	StringTie	19984937	19985225	+	MSTRG.323	transcript_id “MSTRG.323.1”
SM_V10_1	StringTie	21963724	21968857	-	MSTRG.353	transcript_id “MSTRG.353.1”
SM_V10_1	StringTie	21980953	21985388	-	MSTRG.354	transcript_id “MSTRG.354.1”
SM_V10_1	StringTie	22447288	22448659	+	MSTRG.360	transcript_id “MSTRG.360.1”
SM_V10_1	StringTie	22955069	22959177	+	MSTRG.364	transcript_id “MSTRG.364.7”
SM_V10_1	StringTie	23341585	23342120	-	MSTRG.372	transcript_id “MSTRG.372.1”
SM_V10_1	StringTie	23641006	23641373	+	MSTRG.381	transcript_id “MSTRG.381.1”
SM_V10_1	StringTie	24255572	24257757	-	MSTRG.393	transcript_id “MSTRG.393.1”
SM_V10_1	StringTie	24342766	24344320	+	MSTRG.397	transcript_id “MSTRG.397.1”
SM_V10_1	StringTie	24400819	24402544	-	MSTRG.401	transcript_id “MSTRG.401.1”
SM_V10_1	StringTie	24412282	24414578	+	MSTRG.402	transcript_id “MSTRG.402.1”
SM_V10_1	StringTie	25841520	25842940	+	MSTRG.424	transcript_id “MSTRG.424.1”
SM_V10_1	StringTie	26058517	26060747	-	MSTRG.428	transcript_id “MSTRG.428.1”
SM_V10_1	StringTie	26059164	26060791	+	MSTRG.429	transcript_id “MSTRG.429.1”
SM_V10_1	StringTie	26142177	26151970	-	MSTRG.431	transcript_id “MSTRG.431.1”
SM_V10_1	StringTie	26379014	26382565	-	MSTRG.434	transcript_id “MSTRG.434.1”
SM_V10_1	StringTie	26777387	26777643	+	MSTRG.446	transcript_id “MSTRG.446.1”
SM_V10_1	StringTie	26976737	26978493	+	MSTRG.450	transcript_id “MSTRG.450.1”
SM_V10_1	StringTie	26976803	26977123	+	MSTRG.450	transcript_id “MSTRG.450.2”
SM_V10_1	StringTie	27046612	27046929	-	MSTRG.451	transcript_id “MSTRG.451.1”
SM_V10_1	StringTie	28003250	28009218	-	MSTRG.465	transcript_id “MSTRG.465.1”
SM_V10_1	StringTie	28270690	28292419	+	MSTRG.469	transcript_id “MSTRG.469.1”
SM_V10_1	StringTie	28276878	28292419	+	MSTRG.469	transcript_id “MSTRG.469.4”
SM_V10_1	StringTie	28683162	28684328	-	MSTRG.477	transcript_id “MSTRG.477.1”
SM_V10_1	StringTie	28732616	28735258	-	MSTRG.481	transcript_id “MSTRG.481.1”
SM_V10_1	StringTie	28732730	28735219	-	MSTRG.481	transcript_id “MSTRG.481.2”
SM_V10_1	StringTie	28732822	28737769	-	MSTRG.481	transcript_id “MSTRG.481.3”
SM_V10_1	StringTie	28732864	28733319	-	MSTRG.481	transcript_id “MSTRG.481.4”
SM_V10_1	StringTie	28732866	28733319	-	MSTRG.481	transcript_id “MSTRG.481.5”
SM_V10_1	StringTie	28993909	28994539	+	MSTRG.485	transcript_id “MSTRG.485.1”
SM_V10_1	StringTie	29052166	29054451	-	MSTRG.487	transcript_id “MSTRG.487.1”
SM_V10_1	StringTie	29102341	29107358	+	MSTRG.488	transcript_id “MSTRG.488.1”
SM_V10_1	StringTie	29285253	29301958	+	MSTRG.491	transcript_id “MSTRG.491.1”
SM_V10_1	StringTie	29298185	29301958	+	MSTRG.491	transcript_id “MSTRG.491.3”
SM_V10_1	StringTie	29301080	29301958	+	MSTRG.491	transcript_id “MSTRG.491.7”
SM_V10_1	StringTie	29346161	29346808	-	MSTRG.494	transcript_id “MSTRG.494.9”
SM_V10_1	StringTie	29501191	29501871	+	MSTRG.497	transcript_id “MSTRG.497.1”
SM_V10_1	StringTie	29501208	29501871	+	MSTRG.497	transcript_id “MSTRG.497.2”
SM_V10_1	StringTie	30607900	30608189	-	MSTRG.516	transcript_id “MSTRG.516.1”
SM_V10_1	StringTie	31451267	31451763	+	MSTRG.531	transcript_id “MSTRG.531.2”
SM_V10_1	StringTie	31451356	31451763	+	MSTRG.531	transcript_id “MSTRG.531.3”

Chromosme	Source	Start	End	Strand	Gene	Transcript
SM_V10_1	StringTie	31535926	31544713	-	MSTRG.537	transcript_id “MSTRG.537.1”
SM_V10_1	StringTie	32148560	32148803	-	MSTRG.555	transcript_id “MSTRG.555.1”
SM_V10_1	StringTie	32344115	32350173	-	MSTRG.560	transcript_id “MSTRG.560.12”
SM_V10_1	StringTie	32351964	32354709	+	MSTRG.562	transcript_id “MSTRG.562.1”
SM_V10_1	StringTie	32352230	32354971	+	MSTRG.562	transcript_id “MSTRG.562.2”
SM_V10_1	StringTie	32352262	32354864	+	MSTRG.562	transcript_id “MSTRG.562.3”
SM_V10_1	StringTie	32352410	32354668	+	MSTRG.562	transcript_id “MSTRG.562.4”
SM_V10_1	StringTie	32353532	32354709	+	MSTRG.562	transcript_id “MSTRG.562.5”
SM_V10_1	StringTie	32410157	32413001	-	MSTRG.564	transcript_id “MSTRG.564.1”
SM_V10_1	StringTie	32483415	32483928	-	MSTRG.566	transcript_id “MSTRG.566.1”
SM_V10_1	StringTie	33309323	33312456	-	MSTRG.575	transcript_id “MSTRG.575.1”
SM_V10_1	StringTie	33516738	33520417	-	MSTRG.579	transcript_id “MSTRG.579.1”
SM_V10_1	StringTie	33516844	33519937	-	MSTRG.579	transcript_id “MSTRG.579.2”
SM_V10_1	StringTie	33545682	33549884	+	MSTRG.580	transcript_id “MSTRG.580.1”
SM_V10_1	StringTie	33699735	33700858	-	MSTRG.582	transcript_id “MSTRG.582.1”
SM_V10_1	StringTie	33792565	33793308	+	MSTRG.585	transcript_id “MSTRG.585.1”
SM_V10_1	StringTie	33792565	33802692	+	MSTRG.585	transcript_id “MSTRG.585.2”
SM_V10_1	StringTie	33802255	33802692	+	MSTRG.585	transcript_id “MSTRG.585.3”
SM_V10_1	StringTie	33928015	33928321	+	MSTRG.591	transcript_id “MSTRG.591.1”
SM_V10_1	StringTie	34210724	34211149	+	MSTRG.603	transcript_id “MSTRG.603.1”
SM_V10_1	StringTie	34597881	34598812	-	MSTRG.617	transcript_id “MSTRG.617.5”
SM_V10_1	StringTie	34803632	34803949	-	MSTRG.623	transcript_id “MSTRG.623.1”
SM_V10_1	StringTie	35147584	35147956	-	MSTRG.629	transcript_id “MSTRG.629.1”
SM_V10_1	StringTie	35256173	35258000	-	MSTRG.637	transcript_id “MSTRG.637.1”
SM_V10_1	StringTie	35398890	35399306	-	MSTRG.641	transcript_id “MSTRG.641.1”
SM_V10_1	StringTie	36408400	36414446	+	MSTRG.670	transcript_id “MSTRG.670.1”
SM_V10_1	StringTie	36411881	36414446	+	MSTRG.670	transcript_id “MSTRG.670.2”
SM_V10_1	StringTie	37347028	37347272	+	MSTRG.686	transcript_id “MSTRG.686.1”
SM_V10_1	StringTie	38171370	38171636	-	MSTRG.703	transcript_id “MSTRG.703.1”
SM_V10_1	StringTie	38372928	38373369	+	MSTRG.706	transcript_id “MSTRG.706.1”
SM_V10_1	StringTie	38623511	38704951	-	MSTRG.711	transcript_id “MSTRG.711.6”
SM_V10_1	StringTie	38689051	38708928	-	MSTRG.711	transcript_id “MSTRG.711.9”
SM_V10_1	StringTie	38756773	38766940	+	MSTRG.712	transcript_id “MSTRG.712.1”
SM_V10_1	StringTie	39341972	39372934	-	MSTRG.722	transcript_id “MSTRG.722.1”
SM_V10_1	StringTie	39349959	39360804	+	MSTRG.723	transcript_id “MSTRG.723.1”
SM_V10_1	StringTie	39351559	39360804	+	MSTRG.723	transcript_id “MSTRG.723.2”
SM_V10_1	StringTie	39352613	39360804	+	MSTRG.723	transcript_id “MSTRG.723.3”
SM_V10_1	StringTie	39355211	39360804	+	MSTRG.723	transcript_id “MSTRG.723.4”
SM_V10_1	StringTie	39505253	39505541	-	MSTRG.730	transcript_id “MSTRG.730.1”
SM_V10_1	StringTie	39808706	39816564	+	MSTRG.736	transcript_id “MSTRG.736.1”
SM_V10_1	StringTie	40266414	40266698	+	MSTRG.754	transcript_id “MSTRG.754.3”
SM_V10_1	StringTie	40736879	40737882	+	MSTRG.762	transcript_id “MSTRG.762.1”
SM_V10_1	StringTie	40808206	40808530	+	MSTRG.769	transcript_id “MSTRG.769.1”
SM_V10_1	StringTie	40960306	40963783	-	MSTRG.771	transcript_id “MSTRG.771.1”
SM_V10_1	StringTie	41681931	41694806	+	MSTRG.781	transcript_id “MSTRG.781.1”
SM_V10_1	StringTie	41834028	41834612	-	MSTRG.782	transcript_id “MSTRG.782.2”
SM_V10_1	StringTie	41834205	41834612	-	MSTRG.782	transcript_id “MSTRG.782.3”
SM_V10_1	StringTie	42090761	42095191	-	MSTRG.787	transcript_id “MSTRG.787.1”

Chromosme	Source	Start	End	Strand	Gene	Transcript
SM_V10_1	StringTie	42382770	42399225	-	MSTRG.792	transcript_id “MSTRG.792.1”
SM_V10_1	StringTie	42409653	42412523	+	MSTRG.795	transcript_id “MSTRG.795.1”
SM_V10_1	StringTie	42975224	42976116	-	MSTRG.804	transcript_id “MSTRG.804.4”
SM_V10_1	StringTie	42975263	42976116	-	MSTRG.804	transcript_id “MSTRG.804.5”
SM_V10_1	StringTie	43108367	43111556	-	MSTRG.814	transcript_id “MSTRG.814.1”
SM_V10_1	StringTie	43129406	43130337	+	MSTRG.816	transcript_id “MSTRG.816.1”
SM_V10_1	StringTie	43347853	43348161	+	MSTRG.818	transcript_id “MSTRG.818.1”
SM_V10_1	StringTie	43397226	43397841	-	MSTRG.819	transcript_id “MSTRG.819.1”
SM_V10_1	StringTie	43611186	43611855	+	MSTRG.826	transcript_id “MSTRG.826.1”
SM_V10_1	StringTie	43856588	43859064	-	MSTRG.834	transcript_id “MSTRG.834.1”
SM_V10_1	StringTie	43858375	43859064	-	MSTRG.834	transcript_id “MSTRG.834.2”
SM_V10_1	StringTie	43860491	43861618	-	MSTRG.835	transcript_id “MSTRG.835.1”
SM_V10_1	StringTie	43867845	43868105	-	MSTRG.836	transcript_id “MSTRG.836.1”
SM_V10_1	StringTie	44235549	44245142	+	MSTRG.841	transcript_id “MSTRG.841.4”
SM_V10_1	StringTie	44753856	44754275	+	MSTRG.859	transcript_id “MSTRG.859.3”
SM_V10_1	StringTie	44782442	44785019	-	MSTRG.861	transcript_id “MSTRG.861.1”
SM_V10_1	StringTie	44949568	44949945	-	MSTRG.866	transcript_id “MSTRG.866.1”
SM_V10_1	StringTie	45006425	45006928	-	MSTRG.867	transcript_id “MSTRG.867.1”
SM_V10_1	StringTie	45624489	45624901	-	MSTRG.878	transcript_id “MSTRG.878.1”
SM_V10_1	StringTie	46212496	46214665	-	MSTRG.894	transcript_id “MSTRG.894.1”
SM_V10_1	StringTie	47982326	47982997	-	MSTRG.918	transcript_id “MSTRG.918.1”
SM_V10_1	StringTie	47985151	47985401	-	MSTRG.919	transcript_id “MSTRG.919.1”
SM_V10_1	StringTie	48432439	48432738	+	MSTRG.927	transcript_id “MSTRG.927.1”
SM_V10_1	StringTie	49018792	49019071	-	MSTRG.939	transcript_id “MSTRG.939.1”
SM_V10_1	StringTie	49025450	49028284	+	MSTRG.940	transcript_id “MSTRG.940.2”
SM_V10_1	StringTie	49025507	49028163	+	MSTRG.940	transcript_id “MSTRG.940.3”
SM_V10_1	StringTie	49025536	49025963	+	MSTRG.940	transcript_id “MSTRG.940.4”
SM_V10_1	StringTie	49105519	49109061	-	MSTRG.941	transcript_id “MSTRG.941.1”
SM_V10_1	StringTie	49121128	49125330	-	MSTRG.942	transcript_id “MSTRG.942.1”
SM_V10_1	StringTie	49243146	49254344	+	MSTRG.950	transcript_id “MSTRG.950.2”
SM_V10_1	StringTie	49667773	49694288	-	MSTRG.953	transcript_id “MSTRG.953.1”
SM_V10_1	StringTie	49825098	49825664	+	MSTRG.958	transcript_id “MSTRG.958.1”
SM_V10_1	StringTie	50013987	50014751	-	MSTRG.965	transcript_id “MSTRG.965.1”
SM_V10_1	StringTie	50296311	50296813	+	MSTRG.968	transcript_id “MSTRG.968.1”
SM_V10_1	StringTie	50774549	50778055	-	MSTRG.974	transcript_id “MSTRG.974.1”
SM_V10_1	StringTie	50809694	50833890	+	MSTRG.976	transcript_id “MSTRG.976.4”
SM_V10_1	StringTie	50809694	50833890	+	MSTRG.976	transcript_id “MSTRG.976.5”
SM_V10_1	StringTie	50809694	50833890	+	MSTRG.976	transcript_id “MSTRG.976.6”
SM_V10_1	StringTie	50830958	50833890	+	MSTRG.976	transcript_id “MSTRG.976.8”
SM_V10_1	StringTie	50809908	50812473	-	MSTRG.977	transcript_id “MSTRG.977.1”
SM_V10_1	StringTie	50819427	50821456	-	MSTRG.978	transcript_id “MSTRG.978.1”
SM_V10_1	StringTie	50834603	50843184	-	MSTRG.979	transcript_id “MSTRG.979.4”
SM_V10_1	StringTie	50842733	50843184	-	MSTRG.979	transcript_id “MSTRG.979.6”
SM_V10_1	StringTie	50842790	50843184	-	MSTRG.979	transcript_id “MSTRG.979.7”
SM_V10_1	StringTie	50893313	50893990	-	MSTRG.985	transcript_id “MSTRG.985.1”
SM_V10_1	StringTie	50908289	50911055	+	MSTRG.987	transcript_id “MSTRG.987.1”
SM_V10_1	StringTie	51061079	51062637	+	MSTRG.991	transcript_id “MSTRG.991.1”
SM_V10_1	StringTie	51260916	51263507	-	MSTRG.996	transcript_id “MSTRG.996.1”

Chromosme	Source	Start	End	Strand	Gene	Transcript
SM_V10_1	StringTie	51862886	51863265	+	MSTRG.1008	transcript_id “MSTRG.1008.1”
SM_V10_1	StringTie	53577667	53578132	+	MSTRG.1028	transcript_id “MSTRG.1028.1”
SM_V10_1	StringTie	53585907	53586141	+	MSTRG.1029	transcript_id “MSTRG.1029.1”
SM_V10_1	StringTie	55927640	55928327	-	MSTRG.1061	transcript_id “MSTRG.1061.1”
SM_V10_1	StringTie	55931988	55934106	-	MSTRG.1062	transcript_id “MSTRG.1062.1”
SM_V10_1	StringTie	56046812	56048330	-	MSTRG.1063	transcript_id “MSTRG.1063.1”
SM_V10_1	StringTie	56276033	56276871	-	MSTRG.1069	transcript_id “MSTRG.1069.1”
SM_V10_1	StringTie	56547361	56555731	-	MSTRG.1073	transcript_id “MSTRG.1073.1”
SM_V10_1	StringTie	56693181	56694075	+	MSTRG.1078	transcript_id “MSTRG.1078.1”
SM_V10_1	StringTie	56995071	56995450	+	MSTRG.1085	transcript_id “MSTRG.1085.1”
SM_V10_1	StringTie	57583333	57583602	+	MSTRG.1097	transcript_id “MSTRG.1097.1”
SM_V10_1	StringTie	57623175	57625054	-	MSTRG.1100	transcript_id “MSTRG.1100.1”
SM_V10_1	StringTie	58066892	58069855	-	MSTRG.1107	transcript_id “MSTRG.1107.1”
SM_V10_1	StringTie	58143230	58143936	+	MSTRG.1109	transcript_id “MSTRG.1109.1”
SM_V10_1	StringTie	58143230	58143936	+	MSTRG.1109	transcript_id “MSTRG.1109.2”
SM_V10_1	StringTie	58379299	58384101	+	MSTRG.1116	transcript_id “MSTRG.1116.1”
SM_V10_1	StringTie	58514654	58518706	+	MSTRG.1118	transcript_id “MSTRG.1118.1”
SM_V10_1	StringTie	58677011	58677659	-	MSTRG.1120	transcript_id “MSTRG.1120.1”
SM_V10_1	StringTie	58709092	58715265	-	MSTRG.1120	transcript_id “MSTRG.1120.4”
SM_V10_1	StringTie	59888448	59890981	+	MSTRG.1150	transcript_id “MSTRG.1150.1”
SM_V10_1	StringTie	60117285	60119369	-	MSTRG.1155	transcript_id “MSTRG.1155.1”
SM_V10_1	StringTie	60136852	60137105	-	MSTRG.1157	transcript_id “MSTRG.1157.1”
SM_V10_1	StringTie	60167552	60167859	-	MSTRG.1158	transcript_id “MSTRG.1158.1”
SM_V10_1	StringTie	60431067	60433856	-	MSTRG.1159	transcript_id “MSTRG.1159.4”
SM_V10_1	StringTie	60431196	60433594	-	MSTRG.1159	transcript_id “MSTRG.1159.5”
SM_V10_1	StringTie	60575735	60576137	+	MSTRG.1164	transcript_id “MSTRG.1164.1”
SM_V10_1	StringTie	60892526	60893841	+	MSTRG.1170	transcript_id “MSTRG.1170.1”
SM_V10_1	StringTie	60892527	60893841	+	MSTRG.1170	transcript_id “MSTRG.1170.2”
SM_V10_1	StringTie	61113199	61131149	+	MSTRG.1172	transcript_id “MSTRG.1172.1”
SM_V10_1	StringTie	61302323	61307495	-	MSTRG.1177	transcript_id “MSTRG.1177.1”
SM_V10_1	StringTie	61909379	61909926	+	MSTRG.1193	transcript_id “MSTRG.1193.1”
SM_V10_1	StringTie	61925057	61926079	+	MSTRG.1194	transcript_id “MSTRG.1194.2”
SM_V10_1	StringTie	62450702	62451034	+	MSTRG.1207	transcript_id “MSTRG.1207.1”
SM_V10_1	StringTie	63384782	63392820	+	MSTRG.1224	transcript_id “MSTRG.1224.1”
SM_V10_1	StringTie	63490421	63490942	-	MSTRG.1228	transcript_id “MSTRG.1228.1”
SM_V10_1	StringTie	63944113	63947107	-	MSTRG.1230	transcript_id “MSTRG.1230.1”
SM_V10_1	StringTie	64236607	64237573	+	MSTRG.1236	transcript_id “MSTRG.1236.1”
SM_V10_1	StringTie	64236993	64239967	+	MSTRG.1236	transcript_id “MSTRG.1236.2”
SM_V10_1	StringTie	64441970	64442307	-	MSTRG.1241	transcript_id “MSTRG.1241.1”
SM_V10_1	StringTie	64665822	64677123	-	MSTRG.1244	transcript_id “MSTRG.1244.2”
SM_V10_1	StringTie	64666616	64677123	-	MSTRG.1244	transcript_id “MSTRG.1244.3”
SM_V10_1	StringTie	64673973	64677123	-	MSTRG.1244	transcript_id “MSTRG.1244.4”
SM_V10_1	StringTie	64681715	64683155	-	MSTRG.1245	transcript_id “MSTRG.1245.1”
SM_V10_1	StringTie	64683031	64692374	+	MSTRG.1246	transcript_id “MSTRG.1246.1”
SM_V10_1	StringTie	64689972	64692374	+	MSTRG.1246	transcript_id “MSTRG.1246.2”
SM_V10_1	StringTie	64690015	64692374	+	MSTRG.1246	transcript_id “MSTRG.1246.3”
SM_V10_1	StringTie	64691272	64694725	-	MSTRG.1247	transcript_id “MSTRG.1247.1”
SM_V10_1	StringTie	64691971	64694725	-	MSTRG.1247	transcript_id “MSTRG.1247.2”

Chromosme	Source	Start	End	Strand	Gene	Transcript
SM_V10_1	StringTie	64705037	64709022	-	MSTRG.1248	transcript_id “MSTRG.1248.2”
SM_V10_1	StringTie	64707449	64709022	-	MSTRG.1248	transcript_id “MSTRG.1248.3”
SM_V10_1	StringTie	64738441	64738753	-	MSTRG.1250	transcript_id “MSTRG.1250.1”
SM_V10_1	StringTie	64772169	64772593	-	MSTRG.1253	transcript_id “MSTRG.1253.1”
SM_V10_1	StringTie	64854974	64856234	+	MSTRG.1256	transcript_id “MSTRG.1256.1”
SM_V10_1	StringTie	65117601	65124415	-	MSTRG.1266	transcript_id “MSTRG.1266.1”
SM_V10_1	StringTie	65117605	65123279	-	MSTRG.1266	transcript_id “MSTRG.1266.3”
SM_V10_1	StringTie	65119923	65143489	-	MSTRG.1266	transcript_id “MSTRG.1266.6”
SM_V10_1	StringTie	65129863	65143489	-	MSTRG.1266	transcript_id “MSTRG.1266.7”
SM_V10_1	StringTie	65130098	65143489	-	MSTRG.1266	transcript_id “MSTRG.1266.8”
SM_V10_1	StringTie	65142284	65143489	-	MSTRG.1266	transcript_id “MSTRG.1266.10”
SM_V10_1	StringTie	65142400	65143489	-	MSTRG.1266	transcript_id “MSTRG.1266.11”
SM_V10_1	StringTie	65240791	65243579	+	MSTRG.1268	transcript_id “MSTRG.1268.2”
SM_V10_1	StringTie	65295180	65296742	-	MSTRG.1269	transcript_id “MSTRG.1269.1”
SM_V10_1	StringTie	65296443	65296742	-	MSTRG.1269	transcript_id “MSTRG.1269.2”
SM_V10_1	StringTie	66634682	66635752	+	MSTRG.1288	transcript_id “MSTRG.1288.1”
SM_V10_1	StringTie	66703024	66703916	+	MSTRG.1293	transcript_id “MSTRG.1293.1”
SM_V10_1	StringTie	67365518	67366951	-	MSTRG.1306	transcript_id “MSTRG.1306.1”
SM_V10_1	StringTie	68258953	68259261	-	MSTRG.1315	transcript_id “MSTRG.1315.1”
SM_V10_1	StringTie	68270588	68272243	-	MSTRG.1316	transcript_id “MSTRG.1316.1”
SM_V10_1	StringTie	68402169	68402447	+	MSTRG.1322	transcript_id “MSTRG.1322.1”
SM_V10_1	StringTie	69293846	69304430	+	MSTRG.1338	transcript_id “MSTRG.1338.1”
SM_V10_1	StringTie	69643258	69657269	-	MSTRG.1348	transcript_id “MSTRG.1348.1”
SM_V10_1	StringTie	69844608	69845244	+	MSTRG.1357	transcript_id “MSTRG.1357.1”
SM_V10_1	StringTie	70978191	70980862	+	MSTRG.1381	transcript_id “MSTRG.1381.1”
SM_V10_1	StringTie	71411379	71415017	+	MSTRG.1384	transcript_id “MSTRG.1384.5”
SM_V10_1	StringTie	71814010	71814610	+	MSTRG.1388	transcript_id “MSTRG.1388.1”
SM_V10_1	StringTie	71910929	71912211	+	MSTRG.1389	transcript_id “MSTRG.1389.1”
SM_V10_1	StringTie	72164050	72167885	+	MSTRG.1395	transcript_id “MSTRG.1395.1”
SM_V10_1	StringTie	72177428	72177737	+	MSTRG.1397	transcript_id “MSTRG.1397.1”
SM_V10_1	StringTie	72448866	72450323	-	MSTRG.1405	transcript_id “MSTRG.1405.6”
SM_V10_1	StringTie	72449188	72450323	-	MSTRG.1405	transcript_id “MSTRG.1405.8”
SM_V10_1	StringTie	72449747	72450323	-	MSTRG.1405	transcript_id “MSTRG.1405.9”
SM_V10_1	StringTie	74044423	74046127	+	MSTRG.1421	transcript_id “MSTRG.1421.1”
SM_V10_1	StringTie	74689300	74691379	+	MSTRG.1429	transcript_id “MSTRG.1429.1”
SM_V10_1	StringTie	74771925	74781111	+	MSTRG.1432	transcript_id “MSTRG.1432.3”
SM_V10_1	StringTie	74996461	74997367	-	MSTRG.1437	transcript_id “MSTRG.1437.1”
SM_V10_1	StringTie	75743837	75748238	+	MSTRG.1443	transcript_id “MSTRG.1443.1”
SM_V10_1	StringTie	75847624	75848131	+	MSTRG.1446	transcript_id “MSTRG.1446.1”
SM_V10_1	StringTie	76524487	76527189	-	MSTRG.1455	transcript_id “MSTRG.1455.1”
SM_V10_1	StringTie	76986223	76986505	-	MSTRG.1459	transcript_id “MSTRG.1459.1”
SM_V10_1	StringTie	77163356	77164710	-	MSTRG.1464	transcript_id “MSTRG.1464.1”
SM_V10_1	StringTie	77163403	77164710	-	MSTRG.1464	transcript_id “MSTRG.1464.2”
SM_V10_1	StringTie	77163589	77164710	-	MSTRG.1464	transcript_id “MSTRG.1464.3”
SM_V10_1	StringTie	77163589	77164710	-	MSTRG.1464	transcript_id “MSTRG.1464.4”
SM_V10_1	StringTie	77173271	77173636	+	MSTRG.1465	transcript_id “MSTRG.1465.1”
SM_V10_1	StringTie	77200121	77201953	-	MSTRG.1466	transcript_id “MSTRG.1466.1”
SM_V10_1	StringTie	77237791	77239128	-	MSTRG.1468	transcript_id “MSTRG.1468.1”

Chromosme	Source	Start	End	Strand	Gene	Transcript
SM_V10_1	StringTie	77942758	77943083	+	MSTRG.1479	transcript_id "MSTRG.1479.1"
SM_V10_1	StringTie	78065100	78072415	+	MSTRG.1483	transcript_id "MSTRG.1483.1"
SM_V10_1	StringTie	78070924	78072415	+	MSTRG.1483	transcript_id "MSTRG.1483.3"
SM_V10_1	StringTie	78471681	78471927	+	MSTRG.1495	transcript_id "MSTRG.1495.1"
SM_V10_1	StringTie	78570136	78578603	+	MSTRG.1496	transcript_id "MSTRG.1496.1"
SM_V10_1	StringTie	79089753	79090253	-	MSTRG.1504	transcript_id "MSTRG.1504.1"
SM_V10_1	StringTie	79459475	79459820	+	MSTRG.1517	transcript_id "MSTRG.1517.1"
SM_V10_1	StringTie	79908126	79908499	-	MSTRG.1524	transcript_id "MSTRG.1524.1"
SM_V10_1	StringTie	82244457	82246823	-	MSTRG.1555	transcript_id "MSTRG.1555.1"
SM_V10_1	StringTie	82579748	82580181	+	MSTRG.1565	transcript_id "MSTRG.1565.1"
SM_V10_1	StringTie	82772960	82773210	-	MSTRG.1569	transcript_id "MSTRG.1569.1"
SM_V10_1	StringTie	83016221	83043225	-	MSTRG.1571	transcript_id "MSTRG.1571.7"
SM_V10_1	StringTie	83029817	83037872	-	MSTRG.1571	transcript_id "MSTRG.1571.9"
SM_V10_1	StringTie	83034569	83037872	-	MSTRG.1571	transcript_id "MSTRG.1571.12"
SM_V10_1	StringTie	83620984	83621291	-	MSTRG.1583	transcript_id "MSTRG.1583.1"
SM_V10_1	StringTie	87950738	87957307	+	MSTRG.1657	transcript_id "MSTRG.1657.1"
SM_V10_1	StringTie	87951504	87957307	+	MSTRG.1657	transcript_id "MSTRG.1657.2"
SM_V10_2	StringTie	3149183	3149531	-	MSTRG.1748	transcript_id "MSTRG.1748.1"
SM_V10_2	StringTie	3457024	3459667	-	MSTRG.1751	transcript_id "MSTRG.1751.1"
SM_V10_2	StringTie	4255755	4259012	+	MSTRG.1768	transcript_id "MSTRG.1768.1"
SM_V10_2	StringTie	4255755	4259012	+	MSTRG.1768	transcript_id "MSTRG.1768.2"
SM_V10_2	StringTie	4639866	4641728	-	MSTRG.1773	transcript_id "MSTRG.1773.2"
SM_V10_2	StringTie	6029023	6030077	+	MSTRG.1795	transcript_id "MSTRG.1795.1"
SM_V10_2	StringTie	6061254	6063390	-	MSTRG.1796	transcript_id "MSTRG.1796.3"
SM_V10_2	StringTie	6061302	6063390	-	MSTRG.1796	transcript_id "MSTRG.1796.4"
SM_V10_2	StringTie	7977574	7977863	+	MSTRG.1826	transcript_id "MSTRG.1826.1"
SM_V10_2	StringTie	10751054	10751817	+	MSTRG.1864	transcript_id "MSTRG.1864.1"
SM_V10_2	StringTie	11015819	11036107	+	MSTRG.1870	transcript_id "MSTRG.1870.1"
SM_V10_2	StringTie	11354422	11402650	+	MSTRG.1877	transcript_id "MSTRG.1877.1"
SM_V10_2	StringTie	11979543	11980340	-	MSTRG.1883	transcript_id "MSTRG.1883.1"
SM_V10_2	StringTie	11979568	11980063	-	MSTRG.1883	transcript_id "MSTRG.1883.2"
SM_V10_2	StringTie	12132578	12133463	+	MSTRG.1888	transcript_id "MSTRG.1888.1"
SM_V10_2	StringTie	12306362	12310250	-	MSTRG.1891	transcript_id "MSTRG.1891.14"
SM_V10_2	StringTie	12539729	12540404	+	MSTRG.1897	transcript_id "MSTRG.1897.1"
SM_V10_2	StringTie	12702847	12703099	-	MSTRG.1902	transcript_id "MSTRG.1902.1"
SM_V10_2	StringTie	13808451	13810086	+	MSTRG.1924	transcript_id "MSTRG.1924.1"
SM_V10_2	StringTie	13815110	13816515	+	MSTRG.1927	transcript_id "MSTRG.1927.1"
SM_V10_2	StringTie	13953083	13954528	+	MSTRG.1930	transcript_id "MSTRG.1930.1"
SM_V10_2	StringTie	13953409	13954528	+	MSTRG.1930	transcript_id "MSTRG.1930.2"
SM_V10_2	StringTie	13953447	13954528	+	MSTRG.1930	transcript_id "MSTRG.1930.3"
SM_V10_2	StringTie	14045581	14048381	-	MSTRG.1934	transcript_id "MSTRG.1934.1"
SM_V10_2	StringTie	14046217	14048381	-	MSTRG.1934	transcript_id "MSTRG.1934.2"
SM_V10_2	StringTie	14390633	14393454	-	MSTRG.1938	transcript_id "MSTRG.1938.1"
SM_V10_2	StringTie	15265655	15268664	-	MSTRG.1961	transcript_id "MSTRG.1961.1"
SM_V10_2	StringTie	15586458	15588100	+	MSTRG.1969	transcript_id "MSTRG.1969.1"
SM_V10_2	StringTie	15587128	15588100	+	MSTRG.1969	transcript_id "MSTRG.1969.2"
SM_V10_2	StringTie	16142335	16142695	-	MSTRG.1979	transcript_id "MSTRG.1979.1"
SM_V10_2	StringTie	16142442	16142695	-	MSTRG.1979	transcript_id "MSTRG.1979.2"

Chromosme	Source	Start	End	Strand	Gene	Transcript
SM_V10_2	StringTie	16169066	16170035	+	MSTRG.1980	transcript_id “MSTRG.1980.1”
SM_V10_2	StringTie	16290184	16290466	-	MSTRG.1981	transcript_id “MSTRG.1981.1”
SM_V10_2	StringTie	16350200	16353340	+	MSTRG.1982	transcript_id “MSTRG.1982.1”
SM_V10_2	StringTie	17365335	17366085	-	MSTRG.1995	transcript_id “MSTRG.1995.1”
SM_V10_2	StringTie	17371117	17371540	+	MSTRG.1996	transcript_id “MSTRG.1996.1”
SM_V10_2	StringTie	17545274	17546079	+	MSTRG.1999	transcript_id “MSTRG.1999.1”
SM_V10_2	StringTie	17907606	17911489	-	MSTRG.2011	transcript_id “MSTRG.2011.1”
SM_V10_2	StringTie	17975833	17977205	-	MSTRG.2012	transcript_id “MSTRG.2012.1”
SM_V10_2	StringTie	18862621	18868696	-	MSTRG.2029	transcript_id “MSTRG.2029.1”
SM_V10_2	StringTie	18865768	18878422	+	MSTRG.2030	transcript_id “MSTRG.2030.1”
SM_V10_2	StringTie	18932056	18940142	-	MSTRG.2033	transcript_id “MSTRG.2033.1”
SM_V10_2	StringTie	18932146	18940142	-	MSTRG.2033	transcript_id “MSTRG.2033.2”
SM_V10_2	StringTie	18968217	18969189	+	MSTRG.2034	transcript_id “MSTRG.2034.3”
SM_V10_2	StringTie	19671618	19674802	+	MSTRG.2047	transcript_id “MSTRG.2047.2”
SM_V10_2	StringTie	19818080	19818759	-	MSTRG.2053	transcript_id “MSTRG.2053.1”
SM_V10_2	StringTie	19821447	19822894	-	MSTRG.2054	transcript_id “MSTRG.2054.1”
SM_V10_2	StringTie	21543184	21543693	+	MSTRG.2091	transcript_id “MSTRG.2091.5”
SM_V10_2	StringTie	21741257	21741684	-	MSTRG.2095	transcript_id “MSTRG.2095.1”
SM_V10_2	StringTie	21854926	21855508	+	MSTRG.2098	transcript_id “MSTRG.2098.2”
SM_V10_2	StringTie	22311159	22311572	-	MSTRG.2110	transcript_id “MSTRG.2110.1”
SM_V10_2	StringTie	22394129	22396490	+	MSTRG.2111	transcript_id “MSTRG.2111.1”
SM_V10_2	StringTie	23181594	23182475	-	MSTRG.2120	transcript_id “MSTRG.2120.1”
SM_V10_2	StringTie	23507652	23514511	-	MSTRG.2129	transcript_id “MSTRG.2129.1”
SM_V10_2	StringTie	23791772	23795482	-	MSTRG.2131	transcript_id “MSTRG.2131.1”
SM_V10_2	StringTie	23934288	23939600	-	MSTRG.2134	transcript_id “MSTRG.2134.1”
SM_V10_2	StringTie	23980945	23981910	-	MSTRG.2135	transcript_id “MSTRG.2135.2”
SM_V10_2	StringTie	26238417	26241185	-	MSTRG.2181	transcript_id “MSTRG.2181.1”
SM_V10_2	StringTie	26346040	26346312	-	MSTRG.2183	transcript_id “MSTRG.2183.1”
SM_V10_2	StringTie	26806898	26807568	+	MSTRG.2190	transcript_id “MSTRG.2190.1”
SM_V10_2	StringTie	27786818	27787868	+	MSTRG.2194	transcript_id “MSTRG.2194.1”
SM_V10_2	StringTie	28540685	28543922	+	MSTRG.2212	transcript_id “MSTRG.2212.1”
SM_V10_2	StringTie	28792285	28803292	-	MSTRG.2213	transcript_id “MSTRG.2213.1”
SM_V10_2	StringTie	28802977	28803292	-	MSTRG.2213	transcript_id “MSTRG.2213.2”
SM_V10_2	StringTie	28861922	28862282	+	MSTRG.2215	transcript_id “MSTRG.2215.1”
SM_V10_2	StringTie	29174615	29176622	-	MSTRG.2223	transcript_id “MSTRG.2223.1”
SM_V10_2	StringTie	29253045	29254810	+	MSTRG.2225	transcript_id “MSTRG.2225.1”
SM_V10_2	StringTie	29253141	29254810	+	MSTRG.2225	transcript_id “MSTRG.2225.2”
SM_V10_2	StringTie	29825441	29825837	-	MSTRG.2235	transcript_id “MSTRG.2235.1”
SM_V10_2	StringTie	29912794	29913681	-	MSTRG.2240	transcript_id “MSTRG.2240.1”
SM_V10_2	StringTie	29912884	29913681	-	MSTRG.2240	transcript_id “MSTRG.2240.2”
SM_V10_2	StringTie	29913047	29913681	-	MSTRG.2240	transcript_id “MSTRG.2240.3”
SM_V10_2	StringTie	30406262	30415904	+	MSTRG.2244	transcript_id “MSTRG.2244.1”
SM_V10_2	StringTie	30661399	30661837	+	MSTRG.2246	transcript_id “MSTRG.2246.1”
SM_V10_2	StringTie	30779777	30780024	-	MSTRG.2249	transcript_id “MSTRG.2249.1”
SM_V10_2	StringTie	31785867	31787784	-	MSTRG.2277	transcript_id “MSTRG.2277.1”
SM_V10_2	StringTie	31883950	31884586	-	MSTRG.2278	transcript_id “MSTRG.2278.1”
SM_V10_2	StringTie	32287166	32310931	+	MSTRG.2286	transcript_id “MSTRG.2286.1”
SM_V10_2	StringTie	32525069	32525619	-	MSTRG.2289	transcript_id “MSTRG.2289.1”

Chromosme	Source	Start	End	Strand	Gene	Transcript
SM_V10_2	StringTie	33080998	33093007	-	MSTRG.2297	transcript_id “MSTRG.2297.1”
SM_V10_2	StringTie	33093071	33093389	+	MSTRG.2298	transcript_id “MSTRG.2298.1”
SM_V10_2	StringTie	33233540	33233816	+	MSTRG.2302	transcript_id “MSTRG.2302.1”
SM_V10_2	StringTie	33725913	33733443	-	MSTRG.2314	transcript_id “MSTRG.2314.1”
SM_V10_2	StringTie	35027153	35027547	-	MSTRG.2340	transcript_id “MSTRG.2340.1”
SM_V10_2	StringTie	35027183	35027547	-	MSTRG.2340	transcript_id “MSTRG.2340.2”
SM_V10_2	StringTie	35339584	35341393	+	MSTRG.2346	transcript_id “MSTRG.2346.1”
SM_V10_2	StringTie	35465531	35466391	+	MSTRG.2347	transcript_id “MSTRG.2347.2”
SM_V10_2	StringTie	37172178	37173210	+	MSTRG.2375	transcript_id “MSTRG.2375.1”
SM_V10_2	StringTie	37401671	37401910	+	MSTRG.2378	transcript_id “MSTRG.2378.1”
SM_V10_2	StringTie	37857837	37858116	-	MSTRG.2384	transcript_id “MSTRG.2384.1”
SM_V10_2	StringTie	38089140	38099995	+	MSTRG.2385	transcript_id “MSTRG.2385.1”
SM_V10_2	StringTie	38256670	38261008	+	MSTRG.2388	transcript_id “MSTRG.2388.1”
SM_V10_2	StringTie	38790682	38791296	-	MSTRG.2396	transcript_id “MSTRG.2396.1”
SM_V10_2	StringTie	38810175	38819516	-	MSTRG.2397	transcript_id “MSTRG.2397.1”
SM_V10_2	StringTie	39034100	39035822	-	MSTRG.2400	transcript_id “MSTRG.2400.1”
SM_V10_2	StringTie	39038959	39041099	-	MSTRG.2401	transcript_id “MSTRG.2401.1”
SM_V10_2	StringTie	39216371	39216824	+	MSTRG.2405	transcript_id “MSTRG.2405.1”
SM_V10_2	StringTie	39309283	39309674	+	MSTRG.2406	transcript_id “MSTRG.2406.1”
SM_V10_2	StringTie	41213380	41213689	-	MSTRG.2455	transcript_id “MSTRG.2455.1”
SM_V10_2	StringTie	41585007	41585293	+	MSTRG.2461	transcript_id “MSTRG.2461.1”
SM_V10_2	StringTie	43767722	43771715	-	MSTRG.2498	transcript_id “MSTRG.2498.3”
SM_V10_2	StringTie	43924936	43926390	-	MSTRG.2501	transcript_id “MSTRG.2501.1”
SM_V10_2	StringTie	43943704	43945379	-	MSTRG.2502	transcript_id “MSTRG.2502.1”
SM_V10_2	StringTie	43967164	43967649	-	MSTRG.2503	transcript_id “MSTRG.2503.1”
SM_V10_2	StringTie	44070347	44073549	+	MSTRG.2504	transcript_id “MSTRG.2504.1”
SM_V10_2	StringTie	45463805	45464171	+	MSTRG.2527	transcript_id “MSTRG.2527.1”
SM_V10_3	StringTie	23958	33199	-	MSTRG.2537	transcript_id “MSTRG.2537.1”
SM_V10_3	StringTie	32800	33199	-	MSTRG.2537	transcript_id “MSTRG.2537.2”
SM_V10_3	StringTie	2055899	2056950	-	MSTRG.2581	transcript_id “MSTRG.2581.1”
SM_V10_3	StringTie	2065467	2066518	-	MSTRG.2582	transcript_id “MSTRG.2582.1”
SM_V10_3	StringTie	2783793	2784187	+	MSTRG.2590	transcript_id “MSTRG.2590.2”
SM_V10_3	StringTie	2934292	2942039	+	MSTRG.2593	transcript_id “MSTRG.2593.2”
SM_V10_3	StringTie	2937544	2937923	-	MSTRG.2594	transcript_id “MSTRG.2594.1”
SM_V10_3	StringTie	2960389	2961863	+	MSTRG.2595	transcript_id “MSTRG.2595.1”
SM_V10_3	StringTie	4242159	4244952	-	MSTRG.2618	transcript_id “MSTRG.2618.1”
SM_V10_3	StringTie	5135307	5137038	-	MSTRG.2635	transcript_id “MSTRG.2635.1”
SM_V10_3	StringTie	6274036	6275017	+	MSTRG.2656	transcript_id “MSTRG.2656.1”
SM_V10_3	StringTie	7031602	7032078	-	MSTRG.2669	transcript_id “MSTRG.2669.1”
SM_V10_3	StringTie	7088025	7102727	+	MSTRG.2674	transcript_id “MSTRG.2674.1”
SM_V10_3	StringTie	7811587	7812023	+	MSTRG.2681	transcript_id “MSTRG.2681.1”
SM_V10_3	StringTie	7811611	7812023	+	MSTRG.2681	transcript_id “MSTRG.2681.2”
SM_V10_3	StringTie	8853651	8854177	+	MSTRG.2701	transcript_id “MSTRG.2701.1”
SM_V10_3	StringTie	9284963	9285444	+	MSTRG.2718	transcript_id “MSTRG.2718.1”
SM_V10_3	StringTie	9597802	9598148	+	MSTRG.2726	transcript_id “MSTRG.2726.1”
SM_V10_3	StringTie	11426626	11427171	-	MSTRG.2753	transcript_id “MSTRG.2753.1”
SM_V10_3	StringTie	11540062	11546230	+	MSTRG.2755	transcript_id “MSTRG.2755.1”
SM_V10_3	StringTie	11724824	11725065	+	MSTRG.2759	transcript_id “MSTRG.2759.1”

Chromosme	Source	Start	End	Strand	Gene	Transcript
SM_V10_3	StringTie	14452500	14452804	+	MSTRG.2805	transcript_id “MSTRG.2805.1”
SM_V10_3	StringTie	14824963	14838800	-	MSTRG.2813	transcript_id “MSTRG.2813.1”
SM_V10_3	StringTie	14824963	14838800	-	MSTRG.2813	transcript_id “MSTRG.2813.2”
SM_V10_3	StringTie	14824964	14830783	-	MSTRG.2813	transcript_id “MSTRG.2813.3”
SM_V10_3	StringTie	14825014	14838800	-	MSTRG.2813	transcript_id “MSTRG.2813.4”
SM_V10_3	StringTie	14837112	14838800	-	MSTRG.2813	transcript_id “MSTRG.2813.5”
SM_V10_3	StringTie	14975680	14993031	+	MSTRG.2819	transcript_id “MSTRG.2819.1”
SM_V10_3	StringTie	15146070	15146866	+	MSTRG.2823	transcript_id “MSTRG.2823.1”
SM_V10_3	StringTie	16417387	16420879	+	MSTRG.2846	transcript_id “MSTRG.2846.1”
SM_V10_3	StringTie	16724956	16726364	+	MSTRG.2854	transcript_id “MSTRG.2854.1”
SM_V10_3	StringTie	16725166	16726364	+	MSTRG.2854	transcript_id “MSTRG.2854.2”
SM_V10_3	StringTie	17853721	17858265	+	MSTRG.2870	transcript_id “MSTRG.2870.1”
SM_V10_3	StringTie	18027708	18030248	-	MSTRG.2875	transcript_id “MSTRG.2875.1”
SM_V10_3	StringTie	18208519	18209899	-	MSTRG.2880	transcript_id “MSTRG.2880.1”
SM_V10_3	StringTie	18208838	18209115	-	MSTRG.2880	transcript_id “MSTRG.2880.3”
SM_V10_3	StringTie	18251477	18252131	+	MSTRG.2882	transcript_id “MSTRG.2882.1”
SM_V10_3	StringTie	18828104	18829655	-	MSTRG.2888	transcript_id “MSTRG.2888.1”
SM_V10_3	StringTie	19138864	19139973	+	MSTRG.2890	transcript_id “MSTRG.2890.1”
SM_V10_3	StringTie	20104987	20105292	+	MSTRG.2904	transcript_id “MSTRG.2904.1”
SM_V10_3	StringTie	20177772	20180285	-	MSTRG.2906	transcript_id “MSTRG.2906.1”
SM_V10_3	StringTie	20177802	20180285	-	MSTRG.2906	transcript_id “MSTRG.2906.2”
SM_V10_3	StringTie	20378145	20388401	+	MSTRG.2911	transcript_id “MSTRG.2911.1”
SM_V10_3	StringTie	20382827	20388401	+	MSTRG.2911	transcript_id “MSTRG.2911.2”
SM_V10_3	StringTie	20401481	20402969	+	MSTRG.2912	transcript_id “MSTRG.2912.1”
SM_V10_3	StringTie	21516078	21519447	-	MSTRG.2936	transcript_id “MSTRG.2936.1”
SM_V10_3	StringTie	21593266	21601608	-	MSTRG.2938	transcript_id “MSTRG.2938.1”
SM_V10_3	StringTie	21593320	21598118	-	MSTRG.2938	transcript_id “MSTRG.2938.2”
SM_V10_3	StringTie	21593376	21597456	-	MSTRG.2938	transcript_id “MSTRG.2938.3”
SM_V10_3	StringTie	21597007	21597311	-	MSTRG.2938	transcript_id “MSTRG.2938.4”
SM_V10_3	StringTie	21600683	21601608	-	MSTRG.2938	transcript_id “MSTRG.2938.5”
SM_V10_3	StringTie	21594195	21594902	-	MSTRG.2939	transcript_id “MSTRG.2939.1”
SM_V10_3	StringTie	21594509	21595097	-	MSTRG.2939	transcript_id “MSTRG.2939.2”
SM_V10_3	StringTie	22271360	22276530	+	MSTRG.2949	transcript_id “MSTRG.2949.1”
SM_V10_3	StringTie	22335613	22364031	-	MSTRG.2950	transcript_id “MSTRG.2950.1”
SM_V10_3	StringTie	22445274	22445937	+	MSTRG.2953	transcript_id “MSTRG.2953.1”
SM_V10_3	StringTie	22949000	22953811	-	MSTRG.2961	transcript_id “MSTRG.2961.6”
SM_V10_3	StringTie	22969857	22970346	+	MSTRG.2962	transcript_id “MSTRG.2962.1”
SM_V10_3	StringTie	25195130	25199251	-	MSTRG.2985	transcript_id “MSTRG.2985.1”
SM_V10_3	StringTie	25260552	25261274	-	MSTRG.2987	transcript_id “MSTRG.2987.1”
SM_V10_3	StringTie	25260552	25261274	-	MSTRG.2987	transcript_id “MSTRG.2987.2”
SM_V10_3	StringTie	25260744	25261274	-	MSTRG.2987	transcript_id “MSTRG.2987.3”
SM_V10_3	StringTie	25260792	25261274	-	MSTRG.2987	transcript_id “MSTRG.2987.4”
SM_V10_3	StringTie	25320296	25328880	+	MSTRG.2989	transcript_id “MSTRG.2989.3”
SM_V10_3	StringTie	25326357	25328880	+	MSTRG.2989	transcript_id “MSTRG.2989.5”
SM_V10_3	StringTie	25413894	25414126	-	MSTRG.2991	transcript_id “MSTRG.2991.1”
SM_V10_3	StringTie	25519277	25522950	-	MSTRG.2992	transcript_id “MSTRG.2992.3”
SM_V10_3	StringTie	25519315	25520471	-	MSTRG.2992	transcript_id “MSTRG.2992.4”
SM_V10_3	StringTie	25883652	25884483	+	MSTRG.2997	transcript_id “MSTRG.2997.1”

Chromosme	Source	Start	End	Strand	Gene	Transcript
SM_V10_3	StringTie	26366600	26366876	+	MSTRG.3011	transcript_id “MSTRG.3011.1”
SM_V10_3	StringTie	26522408	26522968	+	MSTRG.3017	transcript_id “MSTRG.3017.1”
SM_V10_3	StringTie	26895459	26898062	+	MSTRG.3025	transcript_id “MSTRG.3025.1”
SM_V10_3	StringTie	27817028	27821351	+	MSTRG.3041	transcript_id “MSTRG.3041.1”
SM_V10_3	StringTie	28458015	28458898	-	MSTRG.3057	transcript_id “MSTRG.3057.1”
SM_V10_3	StringTie	28518171	28523397	-	MSTRG.3059	transcript_id “MSTRG.3059.2”
SM_V10_3	StringTie	28544179	28544438	-	MSTRG.3060	transcript_id “MSTRG.3060.1”
SM_V10_3	StringTie	30103851	30107655	-	MSTRG.3090	transcript_id “MSTRG.3090.1”
SM_V10_3	StringTie	30466625	30469220	+	MSTRG.3095	transcript_id “MSTRG.3095.1”
SM_V10_3	StringTie	30466775	30469220	+	MSTRG.3095	transcript_id “MSTRG.3095.2”
SM_V10_3	StringTie	30466781	30469220	+	MSTRG.3095	transcript_id “MSTRG.3095.3”
SM_V10_3	StringTie	30501465	30504835	-	MSTRG.3096	transcript_id “MSTRG.3096.1”
SM_V10_3	StringTie	30508510	30510726	-	MSTRG.3097	transcript_id “MSTRG.3097.1”
SM_V10_3	StringTie	30957944	30960352	-	MSTRG.3105	transcript_id “MSTRG.3105.1”
SM_V10_3	StringTie	31058084	31061363	+	MSTRG.3106	transcript_id “MSTRG.3106.1”
SM_V10_3	StringTie	31600609	31608594	+	MSTRG.3111	transcript_id “MSTRG.3111.1”
SM_V10_3	StringTie	31719482	31719763	+	MSTRG.3114	transcript_id “MSTRG.3114.1”
SM_V10_3	StringTie	32080143	32080841	-	MSTRG.3120	transcript_id “MSTRG.3120.1”
SM_V10_3	StringTie	32080153	32080841	-	MSTRG.3120	transcript_id “MSTRG.3120.2”
SM_V10_3	StringTie	33423290	33423955	-	MSTRG.3143	transcript_id “MSTRG.3143.1”
SM_V10_3	StringTie	33507388	33507643	-	MSTRG.3146	transcript_id “MSTRG.3146.1”
SM_V10_3	StringTie	33691766	33694806	-	MSTRG.3151	transcript_id “MSTRG.3151.1”
SM_V10_3	StringTie	33944504	33945226	-	MSTRG.3153	transcript_id “MSTRG.3153.1”
SM_V10_3	StringTie	34196733	34206772	+	MSTRG.3156	transcript_id “MSTRG.3156.1”
SM_V10_3	StringTie	34500977	34505670	+	MSTRG.3168	transcript_id “MSTRG.3168.1”
SM_V10_3	StringTie	34691093	34692186	+	MSTRG.3175	transcript_id “MSTRG.3175.2”
SM_V10_3	StringTie	34698508	34721414	+	MSTRG.3176	transcript_id “MSTRG.3176.1”
SM_V10_3	StringTie	34698508	34708463	+	MSTRG.3176	transcript_id “MSTRG.3176.2”
SM_V10_3	StringTie	34698522	34705808	+	MSTRG.3176	transcript_id “MSTRG.3176.3”
SM_V10_3	StringTie	34856933	34858519	+	MSTRG.3179	transcript_id “MSTRG.3179.1”
SM_V10_3	StringTie	35433979	35434349	-	MSTRG.3185	transcript_id “MSTRG.3185.1”
SM_V10_3	StringTie	36398769	36399211	-	MSTRG.3219	transcript_id “MSTRG.3219.1”
SM_V10_3	StringTie	36398783	36399211	-	MSTRG.3219	transcript_id “MSTRG.3219.2”
SM_V10_3	StringTie	37012498	37016724	-	MSTRG.3233	transcript_id “MSTRG.3233.1”
SM_V10_3	StringTie	37124585	37125965	+	MSTRG.3237	transcript_id “MSTRG.3237.1”
SM_V10_3	StringTie	38238943	38239580	-	MSTRG.3251	transcript_id “MSTRG.3251.1”
SM_V10_3	StringTie	38304699	38305097	+	MSTRG.3255	transcript_id “MSTRG.3255.1”
SM_V10_3	StringTie	39746252	39746513	-	MSTRG.3289	transcript_id “MSTRG.3289.1”
SM_V10_3	StringTie	39894056	39897358	+	MSTRG.3295	transcript_id “MSTRG.3295.13”
SM_V10_3	StringTie	40143270	40143835	-	MSTRG.3299	transcript_id “MSTRG.3299.2”
SM_V10_3	StringTie	40143423	40143835	-	MSTRG.3299	transcript_id “MSTRG.3299.3”
SM_V10_3	StringTie	40486971	40490962	+	MSTRG.3307	transcript_id “MSTRG.3307.2”
SM_V10_3	StringTie	40487026	40490962	+	MSTRG.3307	transcript_id “MSTRG.3307.3”
SM_V10_3	StringTie	40773882	40774332	+	MSTRG.3321	transcript_id “MSTRG.3321.2”
SM_V10_3	StringTie	40952947	40954314	-	MSTRG.3325	transcript_id “MSTRG.3325.1”
SM_V10_3	StringTie	44097079	44097674	+	MSTRG.3369	transcript_id “MSTRG.3369.1”
SM_V10_3	StringTie	45225710	45234799	+	MSTRG.3378	transcript_id “MSTRG.3378.1”
SM_V10_3	StringTie	47159853	47160509	-	MSTRG.3428	transcript_id “MSTRG.3428.2”

Chromosme	Source	Start	End	Strand	Gene	Transcript
SM_V10_3	StringTie	47723496	47724091	-	MSTRG.3433	transcript_id “MSTRG.3433.1”
SM_V10_3	StringTie	47723498	47724016	-	MSTRG.3433	transcript_id “MSTRG.3433.2”
SM_V10_4	StringTie	1102479	1103019	-	MSTRG.3493	transcript_id “MSTRG.3493.1”
SM_V10_4	StringTie	1102483	1103019	-	MSTRG.3493	transcript_id “MSTRG.3493.2”
SM_V10_4	StringTie	1224789	1225188	-	MSTRG.3497	transcript_id “MSTRG.3497.2”
SM_V10_4	StringTie	1743529	1747133	-	MSTRG.3504	transcript_id “MSTRG.3504.1”
SM_V10_4	StringTie	3641999	3643396	+	MSTRG.3535	transcript_id “MSTRG.3535.1”
SM_V10_4	StringTie	5040438	5040684	-	MSTRG.3552	transcript_id “MSTRG.3552.1”
SM_V10_4	StringTie	5173432	5174028	+	MSTRG.3556	transcript_id “MSTRG.3556.1”
SM_V10_4	StringTie	7258424	7258678	-	MSTRG.3572	transcript_id “MSTRG.3572.1”
SM_V10_4	StringTie	7655714	7655965	-	MSTRG.3582	transcript_id “MSTRG.3582.1”
SM_V10_4	StringTie	8092024	8092557	+	MSTRG.3586	transcript_id “MSTRG.3586.2”
SM_V10_4	StringTie	8544066	8544380	-	MSTRG.3594	transcript_id “MSTRG.3594.1”
SM_V10_4	StringTie	8956721	8958485	+	MSTRG.3602	transcript_id “MSTRG.3602.1”
SM_V10_4	StringTie	10972278	10972652	-	MSTRG.3636	transcript_id “MSTRG.3636.1”
SM_V10_4	StringTie	11667379	11667645	+	MSTRG.3649	transcript_id “MSTRG.3649.1”
SM_V10_4	StringTie	12526415	12527975	+	MSTRG.3657	transcript_id “MSTRG.3657.1”
SM_V10_4	StringTie	12827580	12827823	+	MSTRG.3662	transcript_id “MSTRG.3662.1”
SM_V10_4	StringTie	13165193	13167074	-	MSTRG.3669	transcript_id “MSTRG.3669.1”
SM_V10_4	StringTie	13198688	13198964	+	MSTRG.3671	transcript_id “MSTRG.3671.1”
SM_V10_4	StringTie	13339746	13340170	-	MSTRG.3676	transcript_id “MSTRG.3676.1”
SM_V10_4	StringTie	13906563	13908166	-	MSTRG.3685	transcript_id “MSTRG.3685.1”
SM_V10_4	StringTie	14231079	14234190	-	MSTRG.3692	transcript_id “MSTRG.3692.1”
SM_V10_4	StringTie	15058927	15068640	+	MSTRG.3709	transcript_id “MSTRG.3709.1”
SM_V10_4	StringTie	15058954	15068640	+	MSTRG.3709	transcript_id “MSTRG.3709.2”
SM_V10_4	StringTie	15059904	15065921	+	MSTRG.3709	transcript_id “MSTRG.3709.3”
SM_V10_4	StringTie	15067839	15068640	+	MSTRG.3709	transcript_id “MSTRG.3709.4”
SM_V10_4	StringTie	15164158	15167286	-	MSTRG.3715	transcript_id “MSTRG.3715.1”
SM_V10_4	StringTie	15425299	15426127	-	MSTRG.3719	transcript_id “MSTRG.3719.1”
SM_V10_4	StringTie	15425371	15426127	-	MSTRG.3719	transcript_id “MSTRG.3719.2”
SM_V10_4	StringTie	15761259	15761945	+	MSTRG.3722	transcript_id “MSTRG.3722.1”
SM_V10_4	StringTie	16241473	16242439	-	MSTRG.3725	transcript_id “MSTRG.3725.1”
SM_V10_4	StringTie	17226425	17227109	-	MSTRG.3736	transcript_id “MSTRG.3736.1”
SM_V10_4	StringTie	17789485	17795865	+	MSTRG.3742	transcript_id “MSTRG.3742.1”
SM_V10_4	StringTie	17789518	17799250	+	MSTRG.3742	transcript_id “MSTRG.3742.2”
SM_V10_4	StringTie	17795866	17799250	+	MSTRG.3742	transcript_id “MSTRG.3742.3”
SM_V10_4	StringTie	17797150	17799250	+	MSTRG.3742	transcript_id “MSTRG.3742.4”
SM_V10_4	StringTie	17796705	17808180	-	MSTRG.3743	transcript_id “MSTRG.3743.1”
SM_V10_4	StringTie	17798390	17806898	-	MSTRG.3743	transcript_id “MSTRG.3743.2”
SM_V10_4	StringTie	18291973	18293775	-	MSTRG.3752	transcript_id “MSTRG.3752.2”
SM_V10_4	StringTie	18631334	18631727	+	MSTRG.3758	transcript_id “MSTRG.3758.1”
SM_V10_4	StringTie	18666545	18667006	+	MSTRG.3760	transcript_id “MSTRG.3760.1”
SM_V10_4	StringTie	19499227	19500855	-	MSTRG.3771	transcript_id “MSTRG.3771.1”
SM_V10_4	StringTie	19501940	19505553	+	MSTRG.3772	transcript_id “MSTRG.3772.5”
SM_V10_4	StringTie	19502072	19505553	+	MSTRG.3772	transcript_id “MSTRG.3772.6”
SM_V10_4	StringTie	19502074	19505553	+	MSTRG.3772	transcript_id “MSTRG.3772.7”
SM_V10_4	StringTie	19587420	19588141	+	MSTRG.3773	transcript_id “MSTRG.3773.1”
SM_V10_4	StringTie	19681055	19681454	+	MSTRG.3777	transcript_id “MSTRG.3777.1”

Chromosme	Source	Start	End	Strand	Gene	Transcript
SM_V10_4	StringTie	19681585	19681939	+	MSTRG.3778	transcript_id “MSTRG.3778.1”
SM_V10_4	StringTie	20342009	20348793	+	MSTRG.3780	transcript_id “MSTRG.3780.1”
SM_V10_4	StringTie	20457769	20473628	+	MSTRG.3782	transcript_id “MSTRG.3782.2”
SM_V10_4	StringTie	20957217	20964121	+	MSTRG.3784	transcript_id “MSTRG.3784.1”
SM_V10_4	StringTie	21898964	21900440	+	MSTRG.3802	transcript_id “MSTRG.3802.1”
SM_V10_4	StringTie	22319241	22320325	+	MSTRG.3811	transcript_id “MSTRG.3811.1”
SM_V10_4	StringTie	23212177	23214780	+	MSTRG.3826	transcript_id “MSTRG.3826.1”
SM_V10_4	StringTie	23804998	23806395	+	MSTRG.3833	transcript_id “MSTRG.3833.1”
SM_V10_4	StringTie	23809182	23809540	-	MSTRG.3834	transcript_id “MSTRG.3834.1”
SM_V10_4	StringTie	24258680	24264823	-	MSTRG.3840	transcript_id “MSTRG.3840.1”
SM_V10_4	StringTie	24259575	24264924	-	MSTRG.3840	transcript_id “MSTRG.3840.2”
SM_V10_4	StringTie	24381798	24390481	+	MSTRG.3844	transcript_id “MSTRG.3844.1”
SM_V10_4	StringTie	24588845	24589345	+	MSTRG.3845	transcript_id “MSTRG.3845.1”
SM_V10_4	StringTie	24841638	24844431	-	MSTRG.3850	transcript_id “MSTRG.3850.1”
SM_V10_4	StringTie	24844836	24847328	-	MSTRG.3851	transcript_id “MSTRG.3851.1”
SM_V10_4	StringTie	24846961	24852260	-	MSTRG.3851	transcript_id “MSTRG.3851.2”
SM_V10_4	StringTie	24918726	24919287	-	MSTRG.3852	transcript_id “MSTRG.3852.8”
SM_V10_4	StringTie	25292024	25299886	-	MSTRG.3861	transcript_id “MSTRG.3861.1”
SM_V10_4	StringTie	25296453	25303761	+	MSTRG.3862	transcript_id “MSTRG.3862.1”
SM_V10_4	StringTie	25372650	25372932	+	MSTRG.3865	transcript_id “MSTRG.3865.1”
SM_V10_4	StringTie	25403333	25403751	-	MSTRG.3867	transcript_id “MSTRG.3867.1”
SM_V10_4	StringTie	25428058	25428588	+	MSTRG.3870	transcript_id “MSTRG.3870.1”
SM_V10_4	StringTie	25507132	25515200	+	MSTRG.3873	transcript_id “MSTRG.3873.1”
SM_V10_4	StringTie	25514841	25515200	+	MSTRG.3873	transcript_id “MSTRG.3873.2”
SM_V10_4	StringTie	25556108	25559294	+	MSTRG.3875	transcript_id “MSTRG.3875.1”
SM_V10_4	StringTie	25556155	25559294	+	MSTRG.3875	transcript_id “MSTRG.3875.2”
SM_V10_4	StringTie	25556490	25559294	+	MSTRG.3875	transcript_id “MSTRG.3875.3”
SM_V10_4	StringTie	25558007	25559294	+	MSTRG.3875	transcript_id “MSTRG.3875.5”
SM_V10_4	StringTie	25923125	25927614	+	MSTRG.3883	transcript_id “MSTRG.3883.1”
SM_V10_4	StringTie	26279653	26281527	+	MSTRG.3890	transcript_id “MSTRG.3890.1”
SM_V10_4	StringTie	26279743	26281527	+	MSTRG.3890	transcript_id “MSTRG.3890.2”
SM_V10_4	StringTie	26621380	26628016	-	MSTRG.3900	transcript_id “MSTRG.3900.1”
SM_V10_4	StringTie	26794805	26795075	+	MSTRG.3902	transcript_id “MSTRG.3902.1”
SM_V10_4	StringTie	28100311	28107269	-	MSTRG.3916	transcript_id “MSTRG.3916.1”
SM_V10_4	StringTie	28294628	28294939	-	MSTRG.3923	transcript_id “MSTRG.3923.1”
SM_V10_4	StringTie	28301628	28302024	+	MSTRG.3924	transcript_id “MSTRG.3924.1”
SM_V10_4	StringTie	28457840	28462524	-	MSTRG.3927	transcript_id “MSTRG.3927.1”
SM_V10_4	StringTie	28754837	28755220	+	MSTRG.3930	transcript_id “MSTRG.3930.1”
SM_V10_4	StringTie	29150343	29171449	+	MSTRG.3942	transcript_id “MSTRG.3942.1”
SM_V10_4	StringTie	29240786	29241301	+	MSTRG.3944	transcript_id “MSTRG.3944.1”
SM_V10_4	StringTie	29490882	29491165	-	MSTRG.3949	transcript_id “MSTRG.3949.1”
SM_V10_4	StringTie	29702758	29703292	-	MSTRG.3953	transcript_id “MSTRG.3953.2”
SM_V10_4	StringTie	30284917	30286191	-	MSTRG.3965	transcript_id “MSTRG.3965.1”
SM_V10_4	StringTie	30511909	30513767	+	MSTRG.3971	transcript_id “MSTRG.3971.1”
SM_V10_4	StringTie	31813393	31814071	+	MSTRG.3983	transcript_id “MSTRG.3983.1”
SM_V10_4	StringTie	31813457	31813949	+	MSTRG.3983	transcript_id “MSTRG.3983.2”
SM_V10_4	StringTie	31813582	31813949	+	MSTRG.3983	transcript_id “MSTRG.3983.3”
SM_V10_4	StringTie	32499913	32507061	-	MSTRG.4000	transcript_id “MSTRG.4000.1”

Chromosme	Source	Start	End	Strand	Gene	Transcript
SM_V10_4	StringTie	32511622	32512228	-	MSTRG.4001	transcript_id "MSTRG.4001.1"
SM_V10_4	StringTie	32981375	32985047	-	MSTRG.4008	transcript_id "MSTRG.4008.1"
SM_V10_4	StringTie	32997571	32998190	+	MSTRG.4009	transcript_id "MSTRG.4009.1"
SM_V10_4	StringTie	33009923	33010178	-	MSTRG.4010	transcript_id "MSTRG.4010.1"
SM_V10_4	StringTie	33070317	33073297	+	MSTRG.4013	transcript_id "MSTRG.4013.12"
SM_V10_4	StringTie	33327832	33330474	-	MSTRG.4020	transcript_id "MSTRG.4020.1"
SM_V10_4	StringTie	34002531	34009242	-	MSTRG.4032	transcript_id "MSTRG.4032.1"
SM_V10_4	StringTie	34220084	34229293	-	MSTRG.4037	transcript_id "MSTRG.4037.1"
SM_V10_4	StringTie	34280747	34282427	-	MSTRG.4041	transcript_id "MSTRG.4041.1"
SM_V10_4	StringTie	34966021	34969180	-	MSTRG.4055	transcript_id "MSTRG.4055.1"
SM_V10_4	StringTie	35057145	35059177	-	MSTRG.4059	transcript_id "MSTRG.4059.1"
SM_V10_4	StringTie	35069347	35071334	+	MSTRG.4060	transcript_id "MSTRG.4060.1"
SM_V10_4	StringTie	35715531	35715837	+	MSTRG.4073	transcript_id "MSTRG.4073.1"
SM_V10_4	StringTie	35928940	35931320	+	MSTRG.4077	transcript_id "MSTRG.4077.3"
SM_V10_4	StringTie	36244573	36245023	-	MSTRG.4081	transcript_id "MSTRG.4081.1"
SM_V10_4	StringTie	36374526	36377689	+	MSTRG.4084	transcript_id "MSTRG.4084.1"
SM_V10_4	StringTie	36374659	36377689	+	MSTRG.4084	transcript_id "MSTRG.4084.2"
SM_V10_4	StringTie	36374768	36377689	+	MSTRG.4084	transcript_id "MSTRG.4084.3"
SM_V10_4	StringTie	36374909	36377689	+	MSTRG.4084	transcript_id "MSTRG.4084.5"
SM_V10_4	StringTie	37084486	37087192	-	MSTRG.4101	transcript_id "MSTRG.4101.2"
SM_V10_4	StringTie	37084947	37087192	-	MSTRG.4101	transcript_id "MSTRG.4101.3"
SM_V10_4	StringTie	37086346	37086793	-	MSTRG.4101	transcript_id "MSTRG.4101.4"
SM_V10_4	StringTie	37280201	37293748	+	MSTRG.4104	transcript_id "MSTRG.4104.1"
SM_V10_4	StringTie	37539693	37543177	+	MSTRG.4110	transcript_id "MSTRG.4110.1"
SM_V10_4	StringTie	38119445	38121674	-	MSTRG.4121	transcript_id "MSTRG.4121.1"
SM_V10_4	StringTie	38368006	38371138	-	MSTRG.4127	transcript_id "MSTRG.4127.1"
SM_V10_4	StringTie	39466648	39469912	-	MSTRG.4130	transcript_id "MSTRG.4130.1"
SM_V10_4	StringTie	40730218	40731960	+	MSTRG.4150	transcript_id "MSTRG.4150.1"
SM_V10_4	StringTie	40730414	40731960	+	MSTRG.4150	transcript_id "MSTRG.4150.2"
SM_V10_4	StringTie	40996749	40997067	+	MSTRG.4152	transcript_id "MSTRG.4152.1"
SM_V10_4	StringTie	42155212	42155486	+	MSTRG.4166	transcript_id "MSTRG.4166.1"
SM_V10_4	StringTie	45285867	45288919	-	MSTRG.4206	transcript_id "MSTRG.4206.1"
SM_V10_5	StringTie	677349	677594	+	MSTRG.4233	transcript_id "MSTRG.4233.1"
SM_V10_5	StringTie	2349368	2349740	-	MSTRG.4284	transcript_id "MSTRG.4284.1"
SM_V10_5	StringTie	2709851	2713731	-	MSTRG.4294	transcript_id "MSTRG.4294.1"
SM_V10_5	StringTie	4344296	4344823	+	MSTRG.4321	transcript_id "MSTRG.4321.1"
SM_V10_5	StringTie	4855752	4856879	-	MSTRG.4329	transcript_id "MSTRG.4329.1"
SM_V10_5	StringTie	4879643	4885146	+	MSTRG.4331	transcript_id "MSTRG.4331.7"
SM_V10_5	StringTie	5272803	5273292	-	MSTRG.4333	transcript_id "MSTRG.4333.1"
SM_V10_5	StringTie	5291358	5296468	+	MSTRG.4335	transcript_id "MSTRG.4335.1"
SM_V10_5	StringTie	8032353	8032593	+	MSTRG.4371	transcript_id "MSTRG.4371.1"
SM_V10_5	StringTie	8778505	8778832	-	MSTRG.4387	transcript_id "MSTRG.4387.1"
SM_V10_5	StringTie	9833782	9834251	+	MSTRG.4400	transcript_id "MSTRG.4400.1"
SM_V10_5	StringTie	10371137	10371702	+	MSTRG.4408	transcript_id "MSTRG.4408.1"
SM_V10_5	StringTie	10461710	10463872	-	MSTRG.4410	transcript_id "MSTRG.4410.1"
SM_V10_5	StringTie	10607291	10608007	+	MSTRG.4416	transcript_id "MSTRG.4416.1"
SM_V10_5	StringTie	10644757	10648796	-	MSTRG.4418	transcript_id "MSTRG.4418.1"
SM_V10_5	StringTie	11105927	11107003	+	MSTRG.4427	transcript_id "MSTRG.4427.1"

Chromosme	Source	Start	End	Strand	Gene	Transcript
SM_V10_5	StringTie	11218632	11219210	+	MSTRG.4428	transcript_id "MSTRG.4428.1"
SM_V10_5	StringTie	12403404	12403950	-	MSTRG.4438	transcript_id "MSTRG.4438.1"
SM_V10_5	StringTie	12952563	12953246	+	MSTRG.4447	transcript_id "MSTRG.4447.1"
SM_V10_5	StringTie	12952688	12953246	+	MSTRG.4447	transcript_id "MSTRG.4447.2"
SM_V10_5	StringTie	13132176	13132785	-	MSTRG.4451	transcript_id "MSTRG.4451.1"
SM_V10_5	StringTie	13132438	13136440	-	MSTRG.4451	transcript_id "MSTRG.4451.2"
SM_V10_5	StringTie	13636093	13636884	+	MSTRG.4456	transcript_id "MSTRG.4456.1"
SM_V10_5	StringTie	13841355	13842437	-	MSTRG.4460	transcript_id "MSTRG.4460.2"
SM_V10_5	StringTie	14525903	14526801	+	MSTRG.4468	transcript_id "MSTRG.4468.1"
SM_V10_5	StringTie	15648799	15649387	+	MSTRG.4476	transcript_id "MSTRG.4476.1"
SM_V10_5	StringTie	15663731	15664392	-	MSTRG.4477	transcript_id "MSTRG.4477.1"
SM_V10_5	StringTie	15673276	15675634	+	MSTRG.4478	transcript_id "MSTRG.4478.1"
SM_V10_5	StringTie	15751782	15758059	-	MSTRG.4482	transcript_id "MSTRG.4482.1"
SM_V10_5	StringTie	15758777	15759321	-	MSTRG.4483	transcript_id "MSTRG.4483.1"
SM_V10_5	StringTie	16345880	16346513	+	MSTRG.4490	transcript_id "MSTRG.4490.1"
SM_V10_5	StringTie	16878107	16878468	+	MSTRG.4499	transcript_id "MSTRG.4499.1"
SM_V10_5	StringTie	16878626	16879151	+	MSTRG.4500	transcript_id "MSTRG.4500.1"
SM_V10_5	StringTie	18348319	18348965	+	MSTRG.4522	transcript_id "MSTRG.4522.1"
SM_V10_5	StringTie	19017355	19018421	-	MSTRG.4527	transcript_id "MSTRG.4527.1"
SM_V10_5	StringTie	19287675	19295661	-	MSTRG.4531	transcript_id "MSTRG.4531.1"
SM_V10_5	StringTie	20328957	20329603	-	MSTRG.4552	transcript_id "MSTRG.4552.1"
SM_V10_5	StringTie	20969247	20970902	-	MSTRG.4564	transcript_id "MSTRG.4564.1"
SM_V10_5	StringTie	21716124	21718355	-	MSTRG.4573	transcript_id "MSTRG.4573.1"
SM_V10_5	StringTie	21763608	21769831	+	MSTRG.4574	transcript_id "MSTRG.4574.1"
SM_V10_5	StringTie	21763939	21764624	+	MSTRG.4575	transcript_id "MSTRG.4575.1"
SM_V10_5	StringTie	21999092	21999376	+	MSTRG.4579	transcript_id "MSTRG.4579.1"
SM_V10_5	StringTie	23070025	23072956	-	MSTRG.4593	transcript_id "MSTRG.4593.1"
SM_V10_5	StringTie	23486852	23488824	+	MSTRG.4603	transcript_id "MSTRG.4603.1"
SM_V10_6	StringTie	3342961	3343997	-	MSTRG.4663	transcript_id "MSTRG.4663.1"
SM_V10_6	StringTie	3343024	3343997	-	MSTRG.4663	transcript_id "MSTRG.4663.2"
SM_V10_6	StringTie	3885600	3886335	+	MSTRG.4670	transcript_id "MSTRG.4670.1"
SM_V10_6	StringTie	4441898	4442406	-	MSTRG.4678	transcript_id "MSTRG.4678.1"
SM_V10_6	StringTie	4750688	4752062	+	MSTRG.4681	transcript_id "MSTRG.4681.1"
SM_V10_6	StringTie	5648088	5648392	+	MSTRG.4710	transcript_id "MSTRG.4710.1"
SM_V10_6	StringTie	5973896	5989288	-	MSTRG.4711	transcript_id "MSTRG.4711.1"
SM_V10_6	StringTie	5973958	5977965	-	MSTRG.4711	transcript_id "MSTRG.4711.2"
SM_V10_6	StringTie	5973958	5985493	-	MSTRG.4711	transcript_id "MSTRG.4711.3"
SM_V10_6	StringTie	5973958	5985493	-	MSTRG.4711	transcript_id "MSTRG.4711.4"
SM_V10_6	StringTie	5973958	5977965	-	MSTRG.4711	transcript_id "MSTRG.4711.5"
SM_V10_6	StringTie	5973958	6003397	-	MSTRG.4711	transcript_id "MSTRG.4711.6"
SM_V10_6	StringTie	5973969	5977965	-	MSTRG.4711	transcript_id "MSTRG.4711.7"
SM_V10_6	StringTie	5975610	5987934	-	MSTRG.4711	transcript_id "MSTRG.4711.10"
SM_V10_6	StringTie	5985059	5985493	-	MSTRG.4711	transcript_id "MSTRG.4711.11"
SM_V10_6	StringTie	6529506	6537018	+	MSTRG.4720	transcript_id "MSTRG.4720.1"
SM_V10_6	StringTie	6986240	6986574	+	MSTRG.4728	transcript_id "MSTRG.4728.2"
SM_V10_6	StringTie	7255234	7266166	+	MSTRG.4736	transcript_id "MSTRG.4736.1"
SM_V10_6	StringTie	7255386	7255819	+	MSTRG.4736	transcript_id "MSTRG.4736.2"
SM_V10_6	StringTie	7588225	7594773	+	MSTRG.4746	transcript_id "MSTRG.4746.1"

Chromosme	Source	Start	End	Strand	Gene	Transcript
SM_V10_6	StringTie	7619643	7619929	+	MSTRG.4747	transcript_id “MSTRG.4747.1”
SM_V10_6	StringTie	8718325	8718948	-	MSTRG.4769	transcript_id “MSTRG.4769.1”
SM_V10_6	StringTie	8962596	8963524	-	MSTRG.4774	transcript_id “MSTRG.4774.1”
SM_V10_6	StringTie	8984351	8984774	+	MSTRG.4775	transcript_id “MSTRG.4775.1”
SM_V10_6	StringTie	9104719	9104965	-	MSTRG.4780	transcript_id “MSTRG.4780.1”
SM_V10_6	StringTie	9266496	9273791	+	MSTRG.4783	transcript_id “MSTRG.4783.1”
SM_V10_6	StringTie	9450266	9454618	-	MSTRG.4786	transcript_id “MSTRG.4786.1”
SM_V10_6	StringTie	9538197	9541117	+	MSTRG.4791	transcript_id “MSTRG.4791.1”
SM_V10_6	StringTie	9798278	9808063	-	MSTRG.4798	transcript_id “MSTRG.4798.1”
SM_V10_6	StringTie	10031895	10033911	+	MSTRG.4810	transcript_id “MSTRG.4810.1”
SM_V10_6	StringTie	11015556	11016323	+	MSTRG.4829	transcript_id “MSTRG.4829.1”
SM_V10_6	StringTie	11360177	11370988	-	MSTRG.4832	transcript_id “MSTRG.4832.1”
SM_V10_6	StringTie	11360316	11370988	-	MSTRG.4832	transcript_id “MSTRG.4832.2”
SM_V10_6	StringTie	11368930	11370988	-	MSTRG.4832	transcript_id “MSTRG.4832.3”
SM_V10_6	StringTie	11368957	11370988	-	MSTRG.4832	transcript_id “MSTRG.4832.4”
SM_V10_6	StringTie	11484327	11485070	-	MSTRG.4834	transcript_id “MSTRG.4834.1”
SM_V10_6	StringTie	11515864	11520157	+	MSTRG.4835	transcript_id “MSTRG.4835.1”
SM_V10_6	StringTie	11515908	11522007	+	MSTRG.4835	transcript_id “MSTRG.4835.2”
SM_V10_6	StringTie	11796566	11797073	-	MSTRG.4842	transcript_id “MSTRG.4842.1”
SM_V10_6	StringTie	12007190	12009648	+	MSTRG.4848	transcript_id “MSTRG.4848.1”
SM_V10_6	StringTie	12083222	12084909	-	MSTRG.4849	transcript_id “MSTRG.4849.1”
SM_V10_6	StringTie	12515692	12517734	+	MSTRG.4860	transcript_id “MSTRG.4860.1”
SM_V10_6	StringTie	13372545	13382216	-	MSTRG.4873	transcript_id “MSTRG.4873.2”
SM_V10_6	StringTie	14781533	14789177	+	MSTRG.4894	transcript_id “MSTRG.4894.5”
SM_V10_6	StringTie	16654628	16655478	-	MSTRG.4934	transcript_id “MSTRG.4934.26”
SM_V10_6	StringTie	17647326	17647854	+	MSTRG.4953	transcript_id “MSTRG.4953.1”
SM_V10_6	StringTie	18244069	18244333	-	MSTRG.4961	transcript_id “MSTRG.4961.1”
SM_V10_6	StringTie	18647862	18649491	-	MSTRG.4967	transcript_id “MSTRG.4967.1”
SM_V10_6	StringTie	19542053	19550024	+	MSTRG.4990	transcript_id “MSTRG.4990.1”
SM_V10_6	StringTie	19770927	19771562	+	MSTRG.4992	transcript_id “MSTRG.4992.1”
SM_V10_6	StringTie	20494117	20494421	+	MSTRG.5005	transcript_id “MSTRG.5005.2”
SM_V10_6	StringTie	20931340	20931989	+	MSTRG.5015	transcript_id “MSTRG.5015.1”
SM_V10_6	StringTie	21021574	21022040	+	MSTRG.5016	transcript_id “MSTRG.5016.1”
SM_V10_6	StringTie	22976143	22976937	+	MSTRG.5054	transcript_id “MSTRG.5054.1”
SM_V10_7	StringTie	323680	323946	-	MSTRG.5092	transcript_id “MSTRG.5092.1”
SM_V10_7	StringTie	333727	334830	+	MSTRG.5093	transcript_id “MSTRG.5093.2”
SM_V10_7	StringTie	1055987	1056226	-	MSTRG.5105	transcript_id “MSTRG.5105.1”
SM_V10_7	StringTie	1828835	1829169	+	MSTRG.5110	transcript_id “MSTRG.5110.1”
SM_V10_7	StringTie	2525586	2537247	+	MSTRG.5122	transcript_id “MSTRG.5122.1”
SM_V10_7	StringTie	2569085	2570028	+	MSTRG.5123	transcript_id “MSTRG.5123.4”
SM_V10_7	StringTie	2667104	2667364	-	MSTRG.5125	transcript_id “MSTRG.5125.1”
SM_V10_7	StringTie	6109764	6111580	-	MSTRG.5164	transcript_id “MSTRG.5164.1”
SM_V10_7	StringTie	6343084	6345278	-	MSTRG.5167	transcript_id “MSTRG.5167.1”
SM_V10_7	StringTie	6674979	6675288	+	MSTRG.5168	transcript_id “MSTRG.5168.1”
SM_V10_7	StringTie	8381063	8381373	+	MSTRG.5188	transcript_id “MSTRG.5188.1”
SM_V10_7	StringTie	8452154	8452413	-	MSTRG.5191	transcript_id “MSTRG.5191.1”
SM_V10_7	StringTie	8510359	8511808	+	MSTRG.5194	transcript_id “MSTRG.5194.1”
SM_V10_7	StringTie	8591422	8593194	-	MSTRG.5198	transcript_id “MSTRG.5198.2”

Chromosme	Source	Start	End	Strand	Gene	Transcript
SM_V10_7	StringTie	8591659	8593652	-	MSTRG.5198	transcript_id “MSTRG.5198.3”
SM_V10_7	StringTie	8940006	8940859	-	MSTRG.5201	transcript_id “MSTRG.5201.1”
SM_V10_7	StringTie	9175093	9175825	-	MSTRG.5203	transcript_id “MSTRG.5203.1”
SM_V10_7	StringTie	10104220	10104975	-	MSTRG.5222	transcript_id “MSTRG.5222.1”
SM_V10_7	StringTie	10311313	10311630	+	MSTRG.5227	transcript_id “MSTRG.5227.2”
SM_V10_7	StringTie	10305974	10312629	+	MSTRG.5228	transcript_id “MSTRG.5228.1”
SM_V10_7	StringTie	11134625	11139023	+	MSTRG.5237	transcript_id “MSTRG.5237.1”
SM_V10_7	StringTie	11154461	11157596	-	MSTRG.5239	transcript_id “MSTRG.5239.2”
SM_V10_7	StringTie	11575172	11575612	+	MSTRG.5244	transcript_id “MSTRG.5244.1”
SM_V10_7	StringTie	11575190	11575612	+	MSTRG.5244	transcript_id “MSTRG.5244.2”
SM_V10_7	StringTie	11645807	11646234	+	MSTRG.5246	transcript_id “MSTRG.5246.1”
SM_V10_7	StringTie	11692731	11693030	+	MSTRG.5250	transcript_id “MSTRG.5250.1”
SM_V10_7	StringTie	12549207	12550707	+	MSTRG.5264	transcript_id “MSTRG.5264.1”
SM_V10_7	StringTie	12784463	12784760	+	MSTRG.5270	transcript_id “MSTRG.5270.1”
SM_V10_7	StringTie	13168575	13176058	+	MSTRG.5275	transcript_id “MSTRG.5275.1”
SM_V10_7	StringTie	13396389	13406906	+	MSTRG.5281	transcript_id “MSTRG.5281.1”
SM_V10_7	StringTie	13396389	13406906	+	MSTRG.5281	transcript_id “MSTRG.5281.2”
SM_V10_7	StringTie	13396389	13406906	+	MSTRG.5281	transcript_id “MSTRG.5281.3”
SM_V10_7	StringTie	13396389	13406906	+	MSTRG.5281	transcript_id “MSTRG.5281.4”
SM_V10_7	StringTie	13396389	13406906	+	MSTRG.5281	transcript_id “MSTRG.5281.5”
SM_V10_7	StringTie	13448212	13466947	+	MSTRG.5282	transcript_id “MSTRG.5282.1”
SM_V10_7	StringTie	13463281	13466947	+	MSTRG.5282	transcript_id “MSTRG.5282.2”
SM_V10_7	StringTie	13529127	13530720	+	MSTRG.5283	transcript_id “MSTRG.5283.1”
SM_V10_7	StringTie	13594486	13595295	-	MSTRG.5286	transcript_id “MSTRG.5286.2”
SM_V10_7	StringTie	13690319	13691338	+	MSTRG.5287	transcript_id “MSTRG.5287.1”
SM_V10_7	StringTie	13690322	13691338	+	MSTRG.5287	transcript_id “MSTRG.5287.2”
SM_V10_7	StringTie	14352325	14358679	-	MSTRG.5291	transcript_id “MSTRG.5291.1”
SM_V10_7	StringTie	14814885	14815169	-	MSTRG.5298	transcript_id “MSTRG.5298.1”
SM_V10_7	StringTie	15018733	15022067	-	MSTRG.5305	transcript_id “MSTRG.5305.1”
SM_V10_7	StringTie	15018762	15022067	-	MSTRG.5305	transcript_id “MSTRG.5305.2”
SM_V10_7	StringTie	17079053	17080410	+	MSTRG.5330	transcript_id “MSTRG.5330.1”
SM_V10_7	StringTie	18587527	18587990	+	MSTRG.5353	transcript_id “MSTRG.5353.1”
SM_V10_7	StringTie	19028290	19030700	+	MSTRG.5361	transcript_id “MSTRG.5361.1”
SM_V10_7	StringTie	19028290	19030700	+	MSTRG.5361	transcript_id “MSTRG.5361.2”
SM_V10_7	StringTie	19029664	19030700	+	MSTRG.5361	transcript_id “MSTRG.5361.4”
SM_V10_MITO	StringTie	22824	26917	+	MSTRG.5385	transcript_id “MSTRG.5385.1”
SM_V10_MITO	StringTie	25547	26917	+	MSTRG.5385	transcript_id “MSTRG.5385.2”
SM_V10_WSR	StringTie	707973	709846	-	MSTRG.5392	transcript_id “MSTRG.5392.7”
SM_V10_WSR	StringTie	2553765	2554435	+	MSTRG.5410	transcript_id “MSTRG.5410.1”
SM_V10_Z	StringTie	2561316	2562833	+	MSTRG.5457	transcript_id “MSTRG.5457.1”
SM_V10_Z	StringTie	3766184	3766743	-	MSTRG.5470	transcript_id “MSTRG.5470.1”
SM_V10_Z	StringTie	3902537	3905075	-	MSTRG.5474	transcript_id “MSTRG.5474.1”
SM_V10_Z	StringTie	4423052	4423329	-	MSTRG.5483	transcript_id “MSTRG.5483.1”
SM_V10_Z	StringTie	4427101	4429998	-	MSTRG.5484	transcript_id “MSTRG.5484.1”
SM_V10_Z	StringTie	4485004	4486126	-	MSTRG.5486	transcript_id “MSTRG.5486.1”
SM_V10_Z	StringTie	6161300	6162364	-	MSTRG.5510	transcript_id “MSTRG.5510.1”
SM_V10_Z	StringTie	6959872	6963182	+	MSTRG.5519	transcript_id “MSTRG.5519.1”
SM_V10_Z	StringTie	7050462	7061349	+	MSTRG.5520	transcript_id “MSTRG.5520.1”

Chromosme	Source	Start	End	Strand	Gene	Transcript
SM_V10_Z	StringTie	7050493	7061349	+	MSTRG.5520	transcript_id "MSTRG.5520.2"
SM_V10_Z	StringTie	7050580	7061349	+	MSTRG.5520	transcript_id "MSTRG.5520.4"
SM_V10_Z	StringTie	7473941	7484880	+	MSTRG.5524	transcript_id "MSTRG.5524.1"
SM_V10_Z	StringTie	7475954	7484880	+	MSTRG.5524	transcript_id "MSTRG.5524.2"
SM_V10_Z	StringTie	7478182	7484880	+	MSTRG.5524	transcript_id "MSTRG.5524.3"
SM_V10_Z	StringTie	7482638	7484808	+	MSTRG.5524	transcript_id "MSTRG.5524.4"
SM_V10_Z	StringTie	7483181	7484880	+	MSTRG.5524	transcript_id "MSTRG.5524.5"
SM_V10_Z	StringTie	7483204	7484880	+	MSTRG.5524	transcript_id "MSTRG.5524.6"
SM_V10_Z	StringTie	7951969	7958286	-	MSTRG.5531	transcript_id "MSTRG.5531.1"
SM_V10_Z	StringTie	7955986	7958286	-	MSTRG.5531	transcript_id "MSTRG.5531.3"
SM_V10_Z	StringTie	7958502	7958822	+	MSTRG.5532	transcript_id "MSTRG.5532.1"
SM_V10_Z	StringTie	8762815	8765781	+	MSTRG.5543	transcript_id "MSTRG.5543.1"
SM_V10_Z	StringTie	9530769	9531058	+	MSTRG.5556	transcript_id "MSTRG.5556.1"
SM_V10_Z	StringTie	9530769	9531058	+	MSTRG.5556	transcript_id "MSTRG.5556.2"
SM_V10_Z	StringTie	9532524	9536546	-	MSTRG.5557	transcript_id "MSTRG.5557.1"
SM_V10_Z	StringTie	9680159	9697657	-	MSTRG.5564	transcript_id "MSTRG.5564.1"
SM_V10_Z	StringTie	9742009	9743182	+	MSTRG.5568	transcript_id "MSTRG.5568.1"
SM_V10_Z	StringTie	9745790	9762895	+	MSTRG.5570	transcript_id "MSTRG.5570.2"
SM_V10_Z	StringTie	10361763	10362057	+	MSTRG.5574	transcript_id "MSTRG.5574.1"
SM_V10_Z	StringTie	10363899	10364174	+	MSTRG.5575	transcript_id "MSTRG.5575.1"
SM_V10_Z	StringTie	10578743	10579231	-	MSTRG.5581	transcript_id "MSTRG.5581.1"
SM_V10_Z	StringTie	10602418	10608992	-	MSTRG.5582	transcript_id "MSTRG.5582.1"
SM_V10_Z	StringTie	10868311	10871560	-	MSTRG.5585	transcript_id "MSTRG.5585.1"
SM_V10_Z	StringTie	11498436	11503383	+	MSTRG.5599	transcript_id "MSTRG.5599.1"
SM_V10_Z	StringTie	11498989	11509905	+	MSTRG.5599	transcript_id "MSTRG.5599.2"
SM_V10_Z	StringTie	11502724	11503383	+	MSTRG.5599	transcript_id "MSTRG.5599.3"
SM_V10_Z	StringTie	12848489	12848830	+	MSTRG.5624	transcript_id "MSTRG.5624.1"
SM_V10_Z	StringTie	13116271	13126006	-	MSTRG.5632	transcript_id "MSTRG.5632.1"
SM_V10_Z	StringTie	13116281	13126384	-	MSTRG.5632	transcript_id "MSTRG.5632.3"
SM_V10_Z	StringTie	13116290	13120450	-	MSTRG.5632	transcript_id "MSTRG.5632.4"
SM_V10_Z	StringTie	13299438	13300418	+	MSTRG.5639	transcript_id "MSTRG.5639.1"
SM_V10_Z	StringTie	14045865	14046798	+	MSTRG.5651	transcript_id "MSTRG.5651.1"
SM_V10_Z	StringTie	14324912	14330139	-	MSTRG.5654	transcript_id "MSTRG.5654.1"
SM_V10_Z	StringTie	14372239	14373261	+	MSTRG.5655	transcript_id "MSTRG.5655.1"
SM_V10_Z	StringTie	14496685	14497160	+	MSTRG.5658	transcript_id "MSTRG.5658.1"
SM_V10_Z	StringTie	14662392	14664653	+	MSTRG.5661	transcript_id "MSTRG.5661.2"
SM_V10_Z	StringTie	14662614	14664653	+	MSTRG.5661	transcript_id "MSTRG.5661.3"
SM_V10_Z	StringTie	14735678	14736255	+	MSTRG.5662	transcript_id "MSTRG.5662.1"
SM_V10_Z	StringTie	15056616	15057180	-	MSTRG.5665	transcript_id "MSTRG.5665.1"
SM_V10_Z	StringTie	15057469	15066966	+	MSTRG.5666	transcript_id "MSTRG.5666.1"
SM_V10_Z	StringTie	15057698	15058882	+	MSTRG.5666	transcript_id "MSTRG.5666.2"
SM_V10_Z	StringTie	15068541	15074380	+	MSTRG.5666	transcript_id "MSTRG.5666.5"
SM_V10_Z	StringTie	15068645	15069729	+	MSTRG.5666	transcript_id "MSTRG.5666.6"
SM_V10_Z	StringTie	15068727	15074380	+	MSTRG.5666	transcript_id "MSTRG.5666.7"
SM_V10_Z	StringTie	15070511	15074380	+	MSTRG.5666	transcript_id "MSTRG.5666.8"
SM_V10_Z	StringTie	15073596	15074380	+	MSTRG.5666	transcript_id "MSTRG.5666.11"
SM_V10_Z	StringTie	15597723	15597955	+	MSTRG.5673	transcript_id "MSTRG.5673.1"
SM_V10_Z	StringTie	16067121	16067672	+	MSTRG.5685	transcript_id "MSTRG.5685.1"

Chromosme	Source	Start	End	Strand	Gene	Transcript
SM_V10_Z	StringTie	16311169	16311440	-	MSTRG.5687	transcript_id “MSTRG.5687.1”
SM_V10_Z	StringTie	16950913	16951173	+	MSTRG.5701	transcript_id “MSTRG.5701.1”
SM_V10_Z	StringTie	17114945	17115529	+	MSTRG.5703	transcript_id “MSTRG.5703.1”
SM_V10_Z	StringTie	17348207	17348588	-	MSTRG.5710	transcript_id “MSTRG.5710.1”
SM_V10_Z	StringTie	17792608	17796429	-	MSTRG.5717	transcript_id “MSTRG.5717.1”
SM_V10_Z	StringTie	17792639	17797260	-	MSTRG.5717	transcript_id “MSTRG.5717.2”
SM_V10_Z	StringTie	17827385	17829095	-	MSTRG.5719	transcript_id “MSTRG.5719.1”
SM_V10_Z	StringTie	17876697	17877799	-	MSTRG.5721	transcript_id “MSTRG.5721.1”
SM_V10_Z	StringTie	18388897	18389741	-	MSTRG.5732	transcript_id “MSTRG.5732.1”
SM_V10_Z	StringTie	18388986	18389741	-	MSTRG.5732	transcript_id “MSTRG.5732.2”
SM_V10_Z	StringTie	18389840	18390128	+	MSTRG.5733	transcript_id “MSTRG.5733.1”
SM_V10_Z	StringTie	18394863	18395360	-	MSTRG.5734	transcript_id “MSTRG.5734.1”
SM_V10_Z	StringTie	18394958	18395360	-	MSTRG.5734	transcript_id “MSTRG.5734.2”
SM_V10_Z	StringTie	18701743	18710023	-	MSTRG.5738	transcript_id “MSTRG.5738.1”
SM_V10_Z	StringTie	19483988	19485994	+	MSTRG.5748	transcript_id “MSTRG.5748.1”
SM_V10_Z	StringTie	19739576	19753691	-	MSTRG.5752	transcript_id “MSTRG.5752.1”
SM_V10_Z	StringTie	20030916	20032388	-	MSTRG.5756	transcript_id “MSTRG.5756.1”
SM_V10_Z	StringTie	20226539	20230830	-	MSTRG.5758	transcript_id “MSTRG.5758.1”
SM_V10_Z	StringTie	20228244	20230830	-	MSTRG.5758	transcript_id “MSTRG.5758.3”
SM_V10_Z	StringTie	20298978	20299456	+	MSTRG.5761	transcript_id “MSTRG.5761.1”
SM_V10_Z	StringTie	20394520	20400210	+	MSTRG.5762	transcript_id “MSTRG.5762.1”
SM_V10_Z	StringTie	20405313	20405615	+	MSTRG.5763	transcript_id “MSTRG.5763.1”
SM_V10_Z	StringTie	20588119	20589199	+	MSTRG.5768	transcript_id “MSTRG.5768.2”
SM_V10_Z	StringTie	20856485	20856731	-	MSTRG.5775	transcript_id “MSTRG.5775.1”
SM_V10_Z	StringTie	20893347	20897356	+	MSTRG.5777	transcript_id “MSTRG.5777.1”
SM_V10_Z	StringTie	21127293	21131156	+	MSTRG.5779	transcript_id “MSTRG.5779.1”
SM_V10_Z	StringTie	21194357	21203142	+	MSTRG.5781	transcript_id “MSTRG.5781.1”
SM_V10_Z	StringTie	21753780	21757906	-	MSTRG.5793	transcript_id “MSTRG.5793.5”
SM_V10_Z	StringTie	21825567	21827801	+	MSTRG.5794	transcript_id “MSTRG.5794.1”
SM_V10_Z	StringTie	22209506	22211251	-	MSTRG.5800	transcript_id “MSTRG.5800.1”
SM_V10_Z	StringTie	22235249	22236120	-	MSTRG.5801	transcript_id “MSTRG.5801.1”
SM_V10_Z	StringTie	22353354	22353889	+	MSTRG.5803	transcript_id “MSTRG.5803.1”
SM_V10_Z	StringTie	22556847	22557916	+	MSTRG.5807	transcript_id “MSTRG.5807.1”
SM_V10_Z	StringTie	22807409	22807817	-	MSTRG.5809	transcript_id “MSTRG.5809.1”
SM_V10_Z	StringTie	22908401	22908666	+	MSTRG.5819	transcript_id “MSTRG.5819.1”
SM_V10_Z	StringTie	23514589	23514877	+	MSTRG.5824	transcript_id “MSTRG.5824.1”
SM_V10_Z	StringTie	23554622	23556012	-	MSTRG.5825	transcript_id “MSTRG.5825.1”
SM_V10_Z	StringTie	23554746	23567426	-	MSTRG.5825	transcript_id “MSTRG.5825.2”
SM_V10_Z	StringTie	23559345	23567426	-	MSTRG.5825	transcript_id “MSTRG.5825.3”
SM_V10_Z	StringTie	23874400	23874849	+	MSTRG.5827	transcript_id “MSTRG.5827.1”
SM_V10_Z	StringTie	23940029	23940522	-	MSTRG.5829	transcript_id “MSTRG.5829.1”
SM_V10_Z	StringTie	24392773	24395041	+	MSTRG.5838	transcript_id “MSTRG.5838.1”
SM_V10_Z	StringTie	24738806	24741935	+	MSTRG.5841	transcript_id “MSTRG.5841.1”
SM_V10_Z	StringTie	24951253	24952217	-	MSTRG.5847	transcript_id “MSTRG.5847.3”
SM_V10_Z	StringTie	25428312	25429336	-	MSTRG.5862	transcript_id “MSTRG.5862.1”
SM_V10_Z	StringTie	25607910	25613631	-	MSTRG.5869	transcript_id “MSTRG.5869.4”
SM_V10_Z	StringTie	25637336	25637629	+	MSTRG.5870	transcript_id “MSTRG.5870.1”
SM_V10_Z	StringTie	25782248	25782636	+	MSTRG.5875	transcript_id “MSTRG.5875.1”

Chromosme	Source	Start	End	Strand	Gene	Transcript
SM_V10_Z	StringTie	26580151	26581519	-	MSTRG.5891	transcript_id "MSTRG.5891.1"
SM_V10_Z	StringTie	26684517	26687176	+	MSTRG.5892	transcript_id "MSTRG.5892.1"
SM_V10_Z	StringTie	26709931	26710826	-	MSTRG.5896	transcript_id "MSTRG.5896.1"
SM_V10_Z	StringTie	27182475	27183613	-	MSTRG.5902	transcript_id "MSTRG.5902.1"
SM_V10_Z	StringTie	27448288	27451237	-	MSTRG.5915	transcript_id "MSTRG.5915.1"
SM_V10_Z	StringTie	27448308	27450176	-	MSTRG.5915	transcript_id "MSTRG.5915.2"
SM_V10_Z	StringTie	27803398	27804009	+	MSTRG.5922	transcript_id "MSTRG.5922.1"
SM_V10_Z	StringTie	27886283	27895324	-	MSTRG.5923	transcript_id "MSTRG.5923.1"
SM_V10_Z	StringTie	27892691	27895324	-	MSTRG.5923	transcript_id "MSTRG.5923.2"
SM_V10_Z	StringTie	28042154	28042443	+	MSTRG.5926	transcript_id "MSTRG.5926.1"
SM_V10_Z	StringTie	28055795	28056524	+	MSTRG.5927	transcript_id "MSTRG.5927.1"
SM_V10_Z	StringTie	28056020	28057894	-	MSTRG.5928	transcript_id "MSTRG.5928.1"
SM_V10_Z	StringTie	28548567	28553080	+	MSTRG.5934	transcript_id "MSTRG.5934.1"
SM_V10_Z	StringTie	28549573	28553080	+	MSTRG.5934	transcript_id "MSTRG.5934.2"
SM_V10_Z	StringTie	28593182	28593946	+	MSTRG.5936	transcript_id "MSTRG.5936.1"
SM_V10_Z	StringTie	28663311	28669829	+	MSTRG.5939	transcript_id "MSTRG.5939.1"
SM_V10_Z	StringTie	28667970	28669829	+	MSTRG.5939	transcript_id "MSTRG.5939.2"
SM_V10_Z	StringTie	28669158	28669580	-	MSTRG.5940	transcript_id "MSTRG.5940.1"
SM_V10_Z	StringTie	29174850	29175901	-	MSTRG.5945	transcript_id "MSTRG.5945.6"
SM_V10_Z	StringTie	29374623	29375049	-	MSTRG.5954	transcript_id "MSTRG.5954.7"
SM_V10_Z	StringTie	30085173	30096373	-	MSTRG.5969	transcript_id "MSTRG.5969.1"
SM_V10_Z	StringTie	30319918	30320314	+	MSTRG.5975	transcript_id "MSTRG.5975.1"
SM_V10_Z	StringTie	30471425	30475959	-	MSTRG.5980	transcript_id "MSTRG.5980.1"
SM_V10_Z	StringTie	31024617	31026392	-	MSTRG.5995	transcript_id "MSTRG.5995.1"
SM_V10_Z	StringTie	31091622	31091862	-	MSTRG.5996	transcript_id "MSTRG.5996.1"
SM_V10_Z	StringTie	31394881	31395919	-	MSTRG.6005	transcript_id "MSTRG.6005.1"
SM_V10_Z	StringTie	31743659	31751118	-	MSTRG.6011	transcript_id "MSTRG.6011.1"
SM_V10_Z	StringTie	32443312	32456313	-	MSTRG.6025	transcript_id "MSTRG.6025.1"
SM_V10_Z	StringTie	33473581	33477076	-	MSTRG.6039	transcript_id "MSTRG.6039.1"
SM_V10_Z	StringTie	35061256	35061904	+	MSTRG.6069	transcript_id "MSTRG.6069.1"
SM_V10_Z	StringTie	35061316	35061904	+	MSTRG.6069	transcript_id "MSTRG.6069.2"
SM_V10_Z	StringTie	35246420	35248179	-	MSTRG.6073	transcript_id "MSTRG.6073.1"
SM_V10_Z	StringTie	35469570	35471918	+	MSTRG.6078	transcript_id "MSTRG.6078.1"
SM_V10_Z	StringTie	35532549	35532920	+	MSTRG.6082	transcript_id "MSTRG.6082.1"
SM_V10_Z	StringTie	35583273	35585470	-	MSTRG.6083	transcript_id "MSTRG.6083.1"
SM_V10_Z	StringTie	37869274	37879161	-	MSTRG.6129	transcript_id "MSTRG.6129.1"
SM_V10_Z	StringTie	37898779	37905709	-	MSTRG.6129	transcript_id "MSTRG.6129.15"
SM_V10_Z	StringTie	37898779	37905709	-	MSTRG.6129	transcript_id "MSTRG.6129.16"
SM_V10_Z	StringTie	41531716	41534389	+	MSTRG.6175	transcript_id "MSTRG.6175.1"
SM_V10_Z	StringTie	42008178	42009949	-	MSTRG.6192	transcript_id "MSTRG.6192.1"
SM_V10_Z	StringTie	42008272	42009949	-	MSTRG.6192	transcript_id "MSTRG.6192.2"
SM_V10_Z	StringTie	42008855	42009949	-	MSTRG.6192	transcript_id "MSTRG.6192.3"
SM_V10_Z	StringTie	42010360	42012695	-	MSTRG.6193	transcript_id "MSTRG.6193.1"
SM_V10_Z	StringTie	42012048	42012695	-	MSTRG.6193	transcript_id "MSTRG.6193.2"
SM_V10_Z	StringTie	42243321	42246396	-	MSTRG.6196	transcript_id "MSTRG.6196.1"
SM_V10_Z	StringTie	42982441	42983366	+	MSTRG.6213	transcript_id "MSTRG.6213.1"
SM_V10_Z	StringTie	43092212	43093171	+	MSTRG.6221	transcript_id "MSTRG.6221.1"
SM_V10_Z	StringTie	43168844	43169612	+	MSTRG.6222	transcript_id "MSTRG.6222.1"

Chromosme	Source	Start	End	Strand	Gene	Transcript
SM_V10_Z	StringTie	43856087	43897594	-	MSTRG.6231	transcript_id “MSTRG.6231.2”
SM_V10_Z	StringTie	43888730	43897951	-	MSTRG.6231	transcript_id “MSTRG.6231.4”
SM_V10_Z	StringTie	44862659	44865568	+	MSTRG.6250	transcript_id “MSTRG.6250.1”
SM_V10_Z	StringTie	44871554	44871821	+	MSTRG.6251	transcript_id “MSTRG.6251.1”
SM_V10_Z	StringTie	45462812	45464998	+	MSTRG.6261	transcript_id “MSTRG.6261.5”
SM_V10_Z	StringTie	45512613	45512916	+	MSTRG.6263	transcript_id “MSTRG.6263.1”
SM_V10_Z	StringTie	46169414	46174146	+	MSTRG.6278	transcript_id “MSTRG.6278.2”
SM_V10_Z	StringTie	46169447	46174146	+	MSTRG.6278	transcript_id “MSTRG.6278.3”
SM_V10_Z	StringTie	46447042	46453094	-	MSTRG.6287	transcript_id “MSTRG.6287.5”
SM_V10_Z	StringTie	46447042	46449473	-	MSTRG.6287	transcript_id “MSTRG.6287.6”
SM_V10_Z	StringTie	46486291	46494292	+	MSTRG.6290	transcript_id “MSTRG.6290.1”
SM_V10_Z	StringTie	47645068	47646042	-	MSTRG.6314	transcript_id “MSTRG.6314.1”
SM_V10_Z	StringTie	47691625	47691871	-	MSTRG.6317	transcript_id “MSTRG.6317.1”
SM_V10_Z	StringTie	47697087	47700299	-	MSTRG.6319	transcript_id “MSTRG.6319.3”
SM_V10_Z	StringTie	47697087	47700299	-	MSTRG.6319	transcript_id “MSTRG.6319.4”
SM_V10_Z	StringTie	47697116	47700299	-	MSTRG.6319	transcript_id “MSTRG.6319.6”
SM_V10_Z	StringTie	47697116	47700299	-	MSTRG.6319	transcript_id “MSTRG.6319.9”
SM_V10_Z	StringTie	47697117	47700299	-	MSTRG.6319	transcript_id “MSTRG.6319.10”
SM_V10_Z	StringTie	47697121	47700299	-	MSTRG.6319	transcript_id “MSTRG.6319.11”
SM_V10_Z	StringTie	47697122	47700299	-	MSTRG.6319	transcript_id “MSTRG.6319.12”
SM_V10_Z	StringTie	47697122	47700299	-	MSTRG.6319	transcript_id “MSTRG.6319.13”
SM_V10_Z	StringTie	47697124	47700299	-	MSTRG.6319	transcript_id “MSTRG.6319.14”
SM_V10_Z	StringTie	47697124	47700299	-	MSTRG.6319	transcript_id “MSTRG.6319.15”
SM_V10_Z	StringTie	47697978	47700299	-	MSTRG.6319	transcript_id “MSTRG.6319.16”
SM_V10_Z	StringTie	47698187	47700299	-	MSTRG.6319	transcript_id “MSTRG.6319.17”
SM_V10_Z	StringTie	47885681	47887985	+	MSTRG.6321	transcript_id “MSTRG.6321.1”
SM_V10_Z	StringTie	47970716	47975624	-	MSTRG.6322	transcript_id “MSTRG.6322.1”
SM_V10_Z	StringTie	47980533	47980927	-	MSTRG.6323	transcript_id “MSTRG.6323.1”
SM_V10_Z	StringTie	48887518	48888029	-	MSTRG.6338	transcript_id “MSTRG.6338.2”
SM_V10_Z	StringTie	49682634	49685610	+	MSTRG.6359	transcript_id “MSTRG.6359.1”
SM_V10_Z	StringTie	49693576	49697002	-	MSTRG.6360	transcript_id “MSTRG.6360.1”
SM_V10_Z	StringTie	49700637	49702478	+	MSTRG.6361	transcript_id “MSTRG.6361.1”
SM_V10_Z	StringTie	49719534	49719940	+	MSTRG.6362	transcript_id “MSTRG.6362.1”
SM_V10_Z	StringTie	50110703	50111071	-	MSTRG.6366	transcript_id “MSTRG.6366.1”
SM_V10_Z	StringTie	50127591	50136997	-	MSTRG.6367	transcript_id “MSTRG.6367.1”
SM_V10_Z	StringTie	50135479	50136997	-	MSTRG.6367	transcript_id “MSTRG.6367.2”
SM_V10_Z	StringTie	50292440	50293850	+	MSTRG.6371	transcript_id “MSTRG.6371.1”
SM_V10_Z	StringTie	50319978	50321653	+	MSTRG.6372	transcript_id “MSTRG.6372.2”
SM_V10_Z	StringTie	50837878	50838120	+	MSTRG.6375	transcript_id “MSTRG.6375.1”
SM_V10_Z	StringTie	50863545	50864395	+	MSTRG.6376	transcript_id “MSTRG.6376.1”
SM_V10_Z	StringTie	50901510	50907696	+	MSTRG.6377	transcript_id “MSTRG.6377.2”
SM_V10_Z	StringTie	51092604	51093455	+	MSTRG.6380	transcript_id “MSTRG.6380.2”
SM_V10_Z	StringTie	51344501	51349472	-	MSTRG.6383	transcript_id “MSTRG.6383.1”
SM_V10_Z	StringTie	52113038	52115935	+	MSTRG.6395	transcript_id “MSTRG.6395.1”
SM_V10_Z	StringTie	52199422	52199676	-	MSTRG.6401	transcript_id “MSTRG.6401.1”
SM_V10_Z	StringTie	52842901	52843308	-	MSTRG.6414	transcript_id “MSTRG.6414.1”
SM_V10_Z	StringTie	52876129	52876828	-	MSTRG.6416	transcript_id “MSTRG.6416.1”
SM_V10_Z	StringTie	52928068	52929174	-	MSTRG.6419	transcript_id “MSTRG.6419.1”

Chromosme	Source	Start	End	Strand	Gene	Transcript
SM_V10_Z	StringTie	53812548	53812932	-	MSTRG.6429	transcript_id "MSTRG.6429.1"
SM_V10_Z	StringTie	54280180	54282974	+	MSTRG.6436	transcript_id "MSTRG.6436.1"
SM_V10_Z	StringTie	54876765	54877049	-	MSTRG.6451	transcript_id "MSTRG.6451.1"
SM_V10_Z	StringTie	54889671	54890489	+	MSTRG.6452	transcript_id "MSTRG.6452.1"
SM_V10_Z	StringTie	54902632	54908201	+	MSTRG.6453	transcript_id "MSTRG.6453.1"
SM_V10_Z	StringTie	54966410	54966671	+	MSTRG.6454	transcript_id "MSTRG.6454.1"
SM_V10_Z	StringTie	56211468	56223085	+	MSTRG.6473	transcript_id "MSTRG.6473.1"
SM_V10_Z	StringTie	56899902	56902059	-	MSTRG.6487	transcript_id "MSTRG.6487.1"
SM_V10_Z	StringTie	56900004	56902059	-	MSTRG.6487	transcript_id "MSTRG.6487.2"
SM_V10_Z	StringTie	56900055	56902059	-	MSTRG.6487	transcript_id "MSTRG.6487.3"
SM_V10_Z	StringTie	57341707	57345751	+	MSTRG.6491	transcript_id "MSTRG.6491.1"
SM_V10_Z	StringTie	58143170	58150527	+	MSTRG.6495	transcript_id "MSTRG.6495.2"
SM_V10_Z	StringTie	58264157	58265397	+	MSTRG.6498	transcript_id "MSTRG.6498.1"
SM_V10_Z	StringTie	58347241	58347702	-	MSTRG.6503	transcript_id "MSTRG.6503.1"
SM_V10_Z	StringTie	58387087	58390758	+	MSTRG.6504	transcript_id "MSTRG.6504.1"
SM_V10_Z	StringTie	58393186	58397055	+	MSTRG.6505	transcript_id "MSTRG.6505.1"
SM_V10_Z	StringTie	58673211	58676832	+	MSTRG.6514	transcript_id "MSTRG.6514.1"
SM_V10_Z	StringTie	58823159	58823606	+	MSTRG.6520	transcript_id "MSTRG.6520.2"
SM_V10_Z	StringTie	58939121	58940439	-	MSTRG.6521	transcript_id "MSTRG.6521.1"
SM_V10_Z	StringTie	58939625	58940439	-	MSTRG.6521	transcript_id "MSTRG.6521.2"
SM_V10_Z	StringTie	58944227	58945523	+	MSTRG.6522	transcript_id "MSTRG.6522.1"
SM_V10_Z	StringTie	58944274	58945523	+	MSTRG.6522	transcript_id "MSTRG.6522.2"
SM_V10_Z	StringTie	58973585	58976280	-	MSTRG.6523	transcript_id "MSTRG.6523.1"
SM_V10_Z	StringTie	58974438	58977226	+	MSTRG.6524	transcript_id "MSTRG.6524.1"
SM_V10_Z	StringTie	58974720	58977226	+	MSTRG.6524	transcript_id "MSTRG.6524.2"
SM_V10_Z	StringTie	58974817	58977226	+	MSTRG.6524	transcript_id "MSTRG.6524.3"
SM_V10_Z	StringTie	59025815	59026574	-	MSTRG.6526	transcript_id "MSTRG.6526.1"
SM_V10_Z	StringTie	59112325	59113033	-	MSTRG.6529	transcript_id "MSTRG.6529.1"
SM_V10_Z	StringTie	60535298	60538871	+	MSTRG.6556	transcript_id "MSTRG.6556.1"
SM_V10_Z	StringTie	60808763	60812419	-	MSTRG.6560	transcript_id "MSTRG.6560.2"
SM_V10_Z	StringTie	60808844	60809437	+	MSTRG.6561	transcript_id "MSTRG.6561.1"
SM_V10_Z	StringTie	60808916	60809437	+	MSTRG.6561	transcript_id "MSTRG.6561.2"
SM_V10_Z	StringTie	60812066	60813496	+	MSTRG.6562	transcript_id "MSTRG.6562.1"
SM_V10_Z	StringTie	60812190	60813496	+	MSTRG.6562	transcript_id "MSTRG.6562.3"
SM_V10_Z	StringTie	60812406	60813496	+	MSTRG.6562	transcript_id "MSTRG.6562.4"
SM_V10_Z	StringTie	61354063	61354446	+	MSTRG.6570	transcript_id "MSTRG.6570.1"
SM_V10_Z	StringTie	62292230	62298863	-	MSTRG.6589	transcript_id "MSTRG.6589.6"
SM_V10_Z	StringTie	62340820	62346783	-	MSTRG.6591	transcript_id "MSTRG.6591.1"
SM_V10_Z	StringTie	62677420	62678466	-	MSTRG.6595	transcript_id "MSTRG.6595.5"
SM_V10_Z	StringTie	62700222	62700490	-	MSTRG.6598	transcript_id "MSTRG.6598.1"
SM_V10_Z	StringTie	62701085	62702967	+	MSTRG.6599	transcript_id "MSTRG.6599.1"
SM_V10_Z	StringTie	62707362	62709115	+	MSTRG.6600	transcript_id "MSTRG.6600.1"
SM_V10_Z	StringTie	62888402	62888724	-	MSTRG.6601	transcript_id "MSTRG.6601.1"
SM_V10_Z	StringTie	63016803	63029062	+	MSTRG.6603	transcript_id "MSTRG.6603.2"
SM_V10_Z	StringTie	63023403	63029062	+	MSTRG.6603	transcript_id "MSTRG.6603.5"
SM_V10_Z	StringTie	63124905	63128654	+	MSTRG.6605	transcript_id "MSTRG.6605.1"
SM_V10_Z	StringTie	63520444	63522320	+	MSTRG.6610	transcript_id "MSTRG.6610.1"
SM_V10_Z	StringTie	63520592	63521295	+	MSTRG.6610	transcript_id "MSTRG.6610.2"

Chromosme	Source	Start	End	Strand	Gene	Transcript
SM_V10_Z	StringTie	63520592	63522320	+	MSTRG.6610	transcript_id “MSTRG.6610.3”
SM_V10_Z	StringTie	63607001	63611898	-	MSTRG.6613	transcript_id “MSTRG.6613.3”
SM_V10_Z	StringTie	63607001	63611898	-	MSTRG.6613	transcript_id “MSTRG.6613.4”
SM_V10_Z	StringTie	64429262	64437114	+	MSTRG.6628	transcript_id “MSTRG.6628.1”
SM_V10_Z	StringTie	65687192	65687800	-	MSTRG.6646	transcript_id “MSTRG.6646.1”
SM_V10_Z	StringTie	67030864	67033074	-	MSTRG.6680	transcript_id “MSTRG.6680.1”
SM_V10_Z	StringTie	67582693	67588407	+	MSTRG.6696	transcript_id “MSTRG.6696.1”
SM_V10_Z	StringTie	67902608	67928961	+	MSTRG.6707	transcript_id “MSTRG.6707.1”
SM_V10_Z	StringTie	68261962	68262215	-	MSTRG.6709	transcript_id “MSTRG.6709.1”
SM_V10_Z	StringTie	68413512	68417405	+	MSTRG.6712	transcript_id “MSTRG.6712.2”
SM_V10_Z	StringTie	68452527	68454409	+	MSTRG.6715	transcript_id “MSTRG.6715.1”
SM_V10_Z	StringTie	70117162	70117760	-	MSTRG.6745	transcript_id “MSTRG.6745.1”
SM_V10_Z	StringTie	70623656	70623979	-	MSTRG.6753	transcript_id “MSTRG.6753.1”
SM_V10_Z	StringTie	70666364	70673663	-	MSTRG.6754	transcript_id “MSTRG.6754.1”
SM_V10_Z	StringTie	71149683	71152546	-	MSTRG.6767	transcript_id “MSTRG.6767.1”
SM_V10_Z	StringTie	72517745	72535925	+	MSTRG.6789	transcript_id “MSTRG.6789.1”
SM_V10_Z	StringTie	72657128	72657537	+	MSTRG.6792	transcript_id “MSTRG.6792.1”
SM_V10_Z	StringTie	72711973	72712361	+	MSTRG.6796	transcript_id “MSTRG.6796.1”
SM_V10_Z	StringTie	72940729	72941186	-	MSTRG.6798	transcript_id “MSTRG.6798.1”
SM_V10_Z	StringTie	73060629	73063546	+	MSTRG.6802	transcript_id “MSTRG.6802.1”
SM_V10_Z	StringTie	73318301	73324005	-	MSTRG.6808	transcript_id “MSTRG.6808.1”
SM_V10_Z	StringTie	73319679	73324005	-	MSTRG.6808	transcript_id “MSTRG.6808.2”
SM_V10_Z	StringTie	74124929	74126982	-	MSTRG.6821	transcript_id “MSTRG.6821.2”
SM_V10_Z	StringTie	77408448	77437419	+	MSTRG.6874	transcript_id “MSTRG.6874.1”
SM_V10_Z	StringTie	78343054	78345322	-	MSTRG.6888	transcript_id “MSTRG.6888.1”
SM_V10_Z	StringTie	78537684	78537942	-	MSTRG.6891	transcript_id “MSTRG.6891.1”
SM_V10_Z	StringTie	79046751	79053075	-	MSTRG.6894	transcript_id “MSTRG.6894.1”
SM_V10_Z	StringTie	80066274	80072390	-	MSTRG.6916	transcript_id “MSTRG.6916.1”
SM_V10_Z	StringTie	81691154	81691412	+	MSTRG.6948	transcript_id “MSTRG.6948.1”
SM_V10_Z	StringTie	86209018	86209552	+	MSTRG.7049	transcript_id “MSTRG.7049.1”

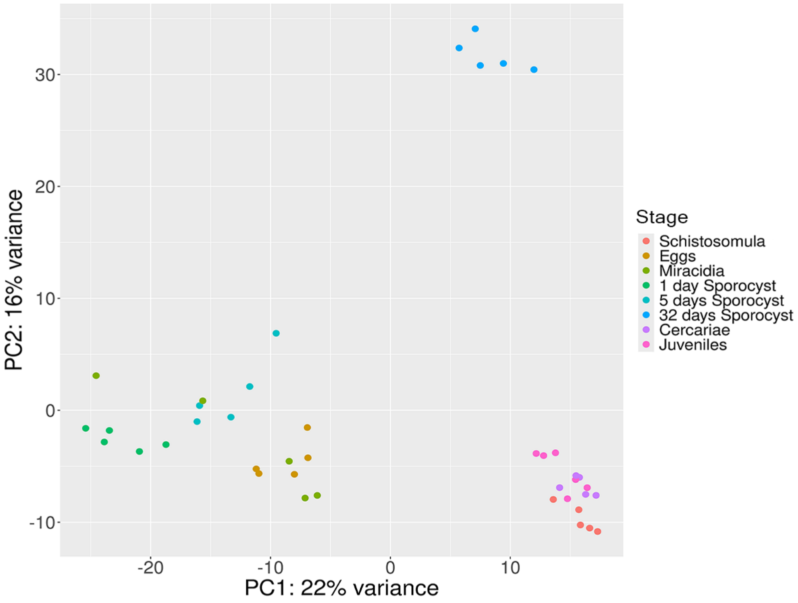

Fig. 1: principal component analysis (PCA) of *Schistosoma mansoni* RNA-seq samples based on vst normalised lncRNA expression values generated in DESeq2. Each point represents a biological replicate, and colours indicate different developmental stages. The first two principal components explain 22% (PC1) and 16% (PC2) of the total variance, respectively, showing clear stage-specific clustering patterns.

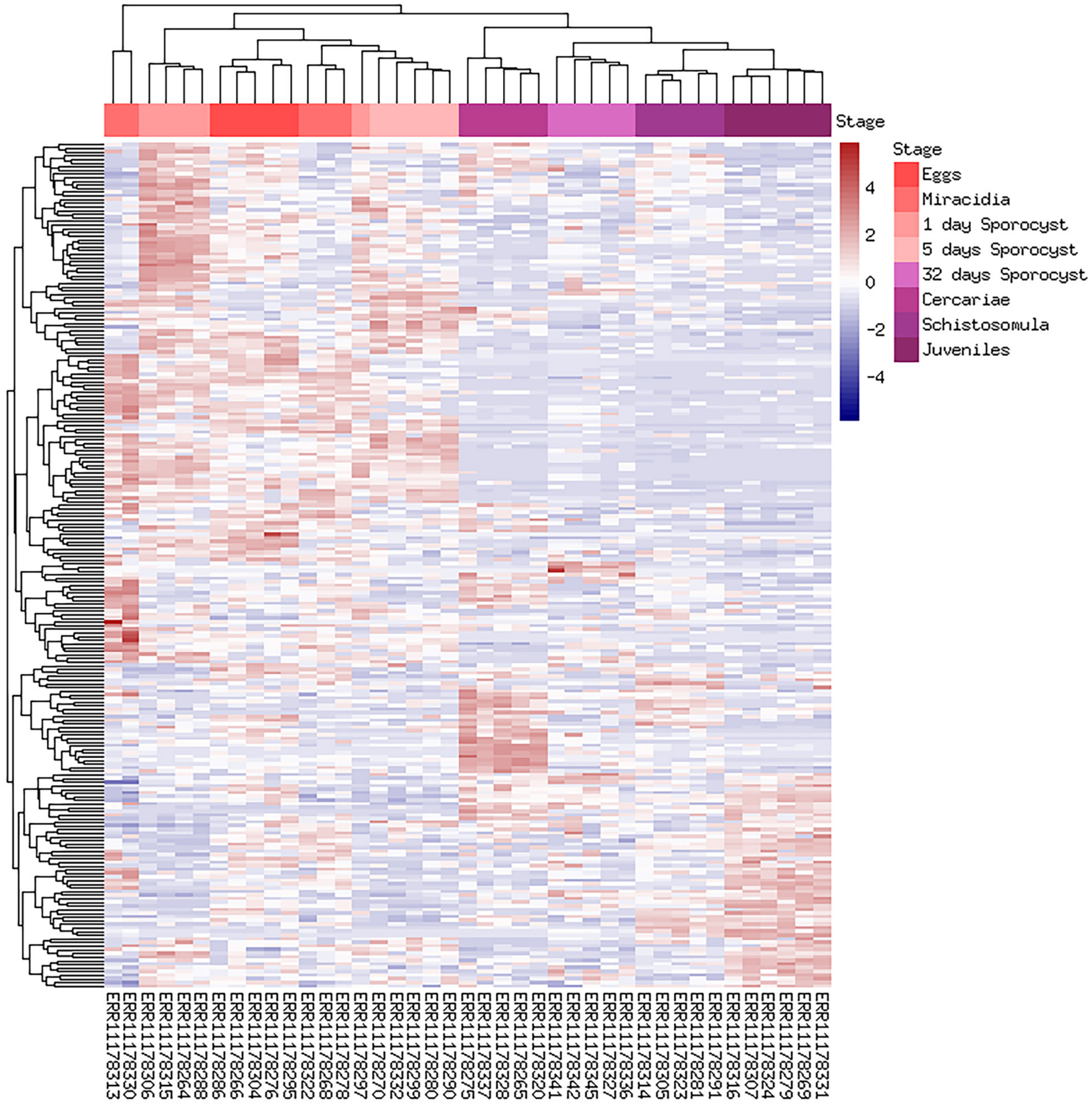

Fig. 2: heatmap of differentially expressed lncRNAs across *Schistosoma mansoni* developmental stages. The heatmap shows the normalized expression (vst) of 1,082 identified lncRNAs obtained with DESeq2. Only transcripts showing significant differential expression ($p < 0.01$) in at least one stage compared to juveniles were included. Colours represent expression values, ranging from low (blue) to high (red) expression. Samples are clustered along the top, with developmental stages indicated by shades of red and violet. Transcripts are hierarchically clustered along the rows.

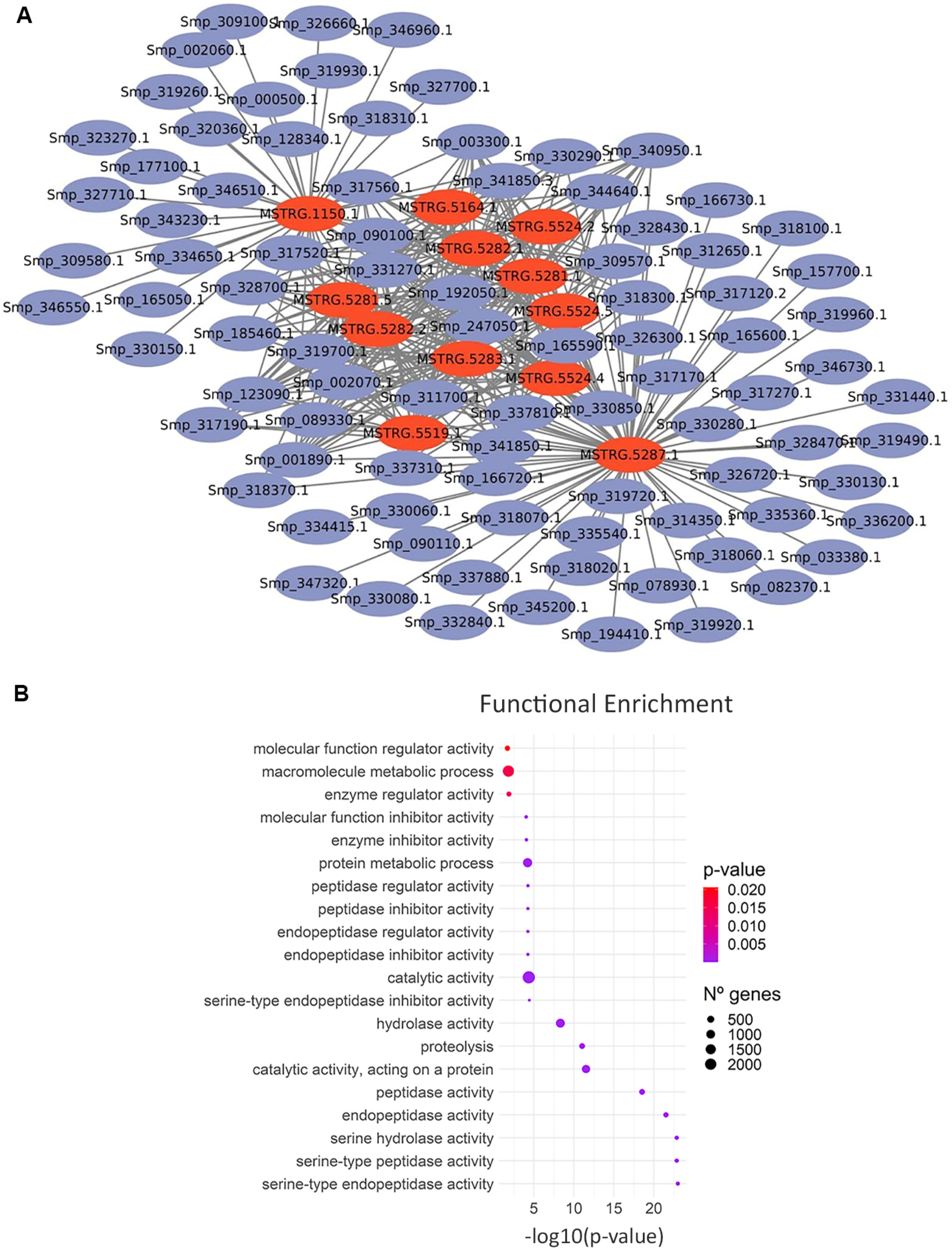

Fig. 3: characterisation of hubs lncRNAs from the magenta module. (A) Subnetwork showing interactions of hub (Module Membership > 0.75 and Gene Significance > 0.5) lncRNAs (red) and their co-expressed mRNAs (blue) from the magenta module. (B) Gene Ontology (GO) enrichment analysis of the co-expressed mRNAs. The y-axis represents the enriched GO terms, while circle size corresponds to the total number of genes associated with each term. The x-axis represents the significance level as -log(p-value), with colours ranging from red (p = 0.020) to violet (p < 0.05).

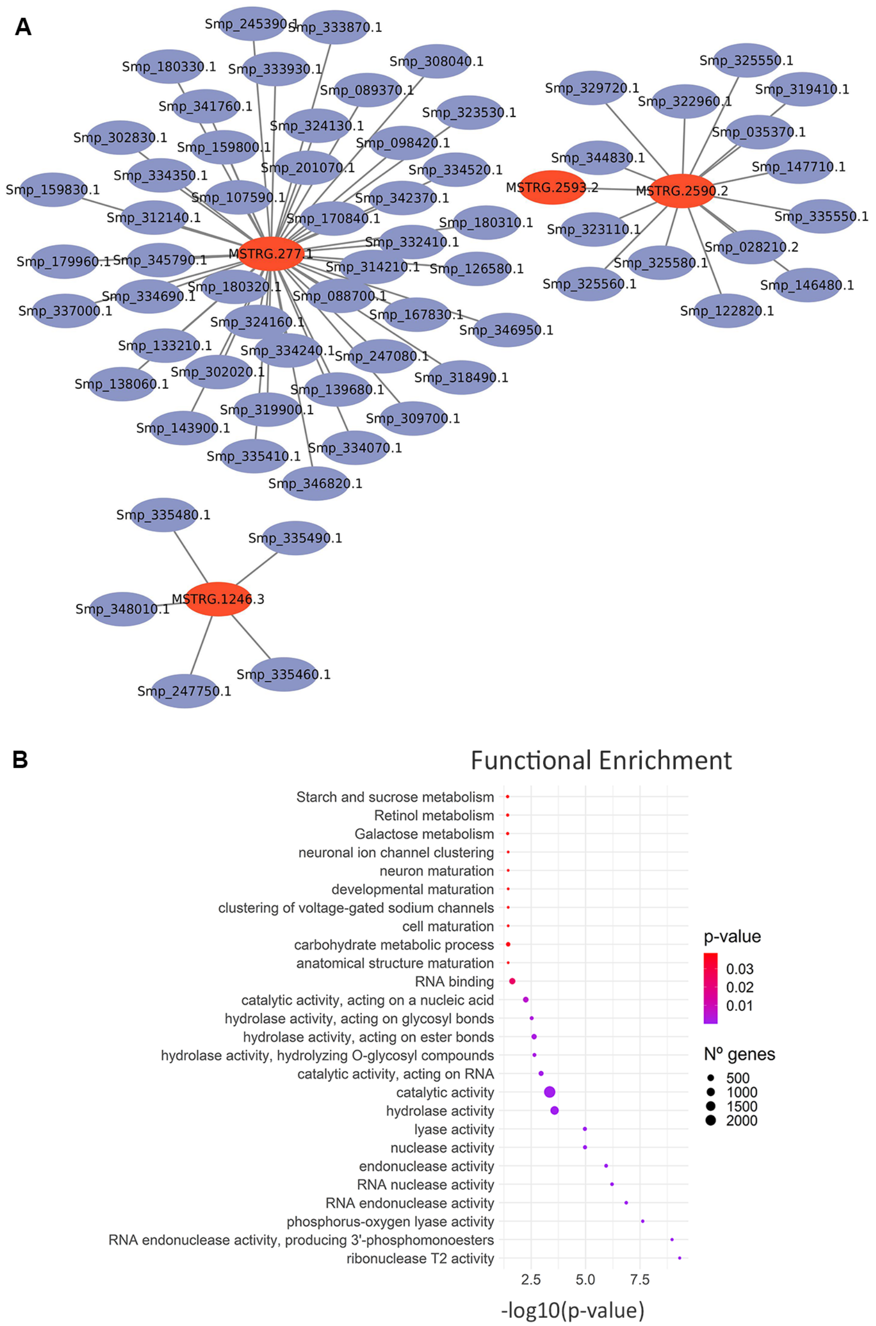

Fig. 4: characterisation of hubs lncRNAs from the black module. (A) Subnetwork showing interactions of hub (Module Membership > 0.75 and Gene Significance > 0.5) lncRNAs (red) and their co-expressed mRNAs (blue) from the black module. (B) Gene Ontology (GO) enrichment analysis of the co-expressed mRNAs. The y-axis represents the enriched GO terms, while circle size corresponds to the total number of genes associated with each term. The x-axis represents the significance level as -log(p-value), with colours ranging from red (p > 0.03) to violet (p < 0.01).

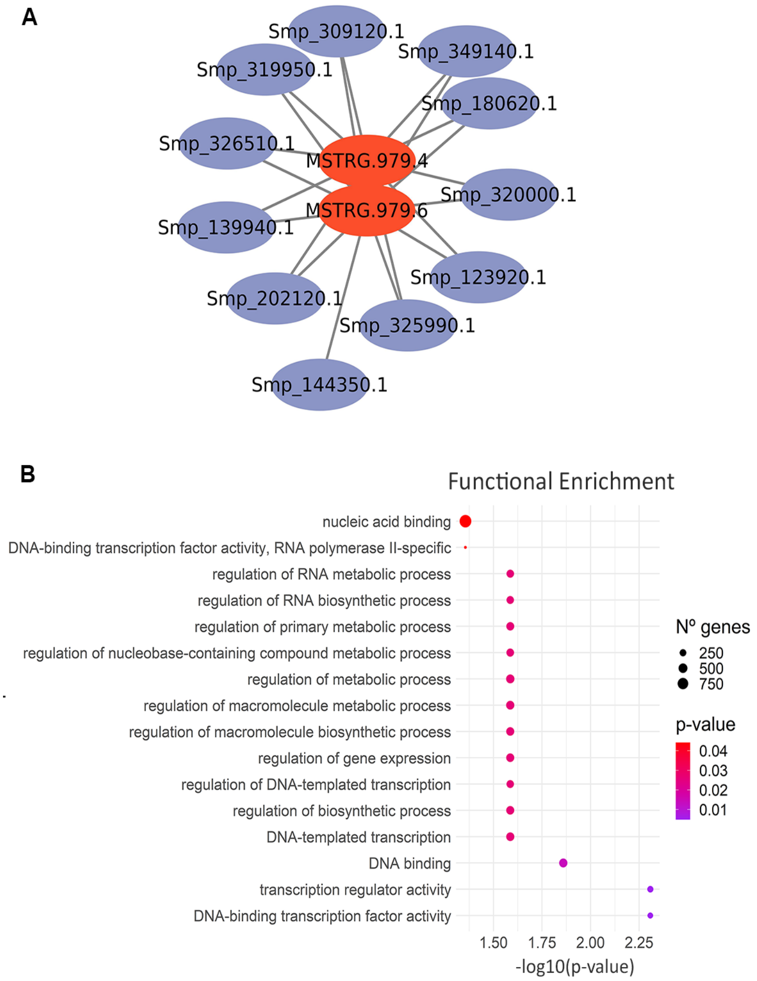

Fig. 5: characterisation of hubs lncRNAs from the salmon module. (A) Subnetwork showing interactions of hub (Module Membership > 0.75 and Gene Significance > 0.5) lncRNAs (red) and their co-expressed mRNAs (blue) from the salmon module. (B) Gene Ontology (GO) enrichment analysis of the co-expressed mRNAs. The y-axis represents the enriched GO terms, while circle size corresponds to the total number of genes associated with each term. The x-axis represents the significance level as -log(p-value), with colours ranging from red (p > 0.04) to violet (p < 0.01).
